# Gloger's Rule or Historical Conjecture? Tests in Mammals

**DOI:** 10.1002/ece3.71855

**Published:** 2025-07-25

**Authors:** Natasha Howell, Tim Caro

**Affiliations:** ^1^ School of Biological Sciences University of Bristol Bristol UK; ^2^ Center for Population Biology University of California Davis California USA

**Keywords:** 19th‐century naturalists, background matching, Constantin Gloger, crypsis, ecogeographical rules, Gloger's rule, mammal coloration

## Abstract

Gloger's rule states that homeotherms are darker at lower latitudes; however, a number of 19th‐century naturalists also suggested that animals are more brightly coloured in the tropics than in temperate regions. Using phylogenetic comparative methods, we investigated and compared both ideas across a global sample of 2726 species of mammals, examining their head, torso, legs and tail regions. Coloration data were obtained from photographs and compared with a colour chart specifically devised for mammals; ecological data were extracted from pre‐existing, open‐source databases. All analyses were conducted using phylogenetic comparative generalised linear mixed models in a Bayesian framework. We found strong support for mammals being darker in the tropics and in areas of high precipitation and evapotranspiration, little support for them being darker in warmer areas, little support for them being redder in more arid regions (a more nuanced interpretation of Gloger's rule), and virtually no support for 19th‐century naturalists' conjecture regarding coloration, contrast, or patterning being more conspicuous in the tropics. These results were replicated at both class and order levels. Our findings provide clear evidence for eumelanic coloration to be more prevalent in more humid climates (one facet of Gloger's rule), operating at a class level, but indicate that 19th‐century observations about bright coloration in the tropics do not pertain to mammals. Our results confirm the importance of Gloger's rule across mammals as a whole and add to a growing tide that darker coloration is linked to humidity at a macroecological scale.

## Graphical abstract

Ecogeographical rules for animal coloration include Gloger's rule, which states that homeotherms are darker at lower latitudes; however, 19th‐century naturalists observed that animals are more colourful in the tropics. We investigated these ideas across the head, torso, legs and tail regions of 2726 species of terrestrial mammals using phylogenetic comparative methods. Our findings provide strong evidence for one facet of Gloger's rule operating at a class level but indicate that 19th‐century observations do not pertain to mammals.

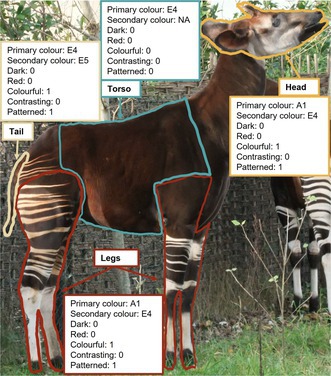

## 1 Introduction

Several ecogeographical rules structure animal and plant morphologies at global scales (Tian and Benton 2020). Bergmann's rule, for example, states that animals are smaller in warmer environments to facilitate efficient heat dissipation and will be larger in colder environments to conserve body heat (Salewski and Watt 2017). Allen's rule, on the other hand, states that animals will have shorter body appendages in colder environments (and vice versa) because smaller surface area to volume ratios on shorter structures are better at conserving heat (Allen 1907).

With regard to animal coloration, Gloger's rule states that within homeothermic species, individuals will be darker at lower latitudes owing to increased melanin deposition in warmer, more humid habitats (the ‘simple’ version of Gloger's rule; Rensch 1929), and this is empirically supported in several taxa, including: rodents (Cerezer et al. 2024), marsupials (Cerezer et al. 2020), pigs (Newell et al. 2021) and squirrels (Sheets and Chavez 2020).

A more complex reinterpretation of Gloger's rule states that environmental temperature and humidity affect different forms of melanin differently. Melanin pigment consists of eumelanin, responsible for grey, brown and black coloration, or phaeomelanin, which produces shades of yellow, orange and red (D'Alba et al. 2014). The complex rule states that eumelanin deposition is greater with increasing humidity and decreases only at very low temperatures, whereas phaeomelanin is manifested in dry and arid conditions with deposition decreasing rapidly at low temperatures (Delhey 2017, 2019). To date, however, rather few attempts have been made to test the complex version in either avian or mammalian taxa, and those that have, such as studies of pigs (Newell et al. 2021), rodents (Cerezer et al. 2024) and ovenbirds (Family Furnariidae; Marcondes et al. 2021) have found mixed results. In the case of mammals, none have examined these at a class level; we attempt to provide such a class‐wide investigation here.

Five suggestions have been proposed to explain the simple version of Gloger's rule: (i) Photoprotection: species living in warmer environments could rely on darker coloration as a form of protection from UV radiation, because highly‐melanised integument has broad light absorption properties that are strong in the spectrum of mutagenic UV light, thereby reducing transmission of UV to the dermis (Jablonski and Chaplin 2000; Nicolaï et al. 2020). (ii) Ectoparasite resistance: melanin inhibits the proliferation of bacterial, fungal and other parasitic infections of the dermis and epidermis (Marcondes et al. 2021). (iii) Melanin protects against mechanical abrasion (Delhey et al. 2023). (iv) Camouflage: dense foliage in the tropics casts dark shadows, and leaf fall generates dark humic leaf litter, thus animals are dark to match their background—this is currently considered the leading causal hypothesis behind Gloger's rule, and has been shown to apply to rodents (Lai et al. 2008) and some families of birds (Miller et al. 2019). (v) Pleiotropy: one of the principal genes regulating melanin‐based colour production, *melanocortin 1 receptor* (*mc1r*), has beneficial pleiotropic effects associated with better anti‐inflammatory, antipyretic and anti‐oxidative responses and is less sensitive to stress which might be beneficial in tropical climes with their higher prevalence of parasites and pathogens (Ducrest et al. 2008).

Bogert's rule, or the thermal melanin hypothesis, runs counter to Gloger's rule, as it links an increase in darkness with greater distance from the equator, rather than closer proximity to it. Specifically, Bogert's rule states that darker animals will be found in colder habitats because darker colours absorb more solar radiation and help animals warm up more quickly, allowing them to start foraging earlier and escape predation more readily earlier in the day and earlier in spring (Bogert 1949). Butterfly species across Europe, for example, conform to Bogert's rule, especially on their basal wings, thorax and abdomen, which are important to thermoregulation (Kang et al. 2021). Bogert's rule applies principally (but perhaps not exclusively) to ectotherms which cannot thermoregulate and therefore will not be considered further here.

A third ecogeographical association comes from conjectural ideas (defined throughout this study as ‘inferences formed without proof or sufficient evidence’) of 19th‐century biologists who wrote much about environmental factors affecting the development of animal coloration and patterning (e.g., Beddard 1895; Wallace 1891). As illustrations, Allen thought that birds had more intense metallic tints towards the tropics; and Scudder remarked that the colours of butterflies become less sharply defined as one travels north; along with many other anecdotal observations of squirrels, carnivores and lepidoptera (see Hingston 1933). Notably, Alfred Russel Wallace investigated the problem in some detail, remarking that the tropics contained ‘an immensely greater number of richly‐coloured birds and insects […] than in temperate and cold countries’ (Wallace 1891, 341), and that ‘local influences [of tropical island localities] seem to favour the production or preservation of intense crimson […] coloration’ (Wallace 1891, 389). This provided further anecdotal support for the existence of a colourfulness gradient skewed towards lower latitudes.

Another separate observation made by Wallace was that more extravagant patterning and ornamentation was ‘most distinctive of [species] which inhabit tropical countries’ (Wallace 1891, 300). Wallace further described ‘contrasted bands’ of colour as being more likely to be observed in tropical localities (Wallace 1891, 273). He argued that a greater diversity of animal appearances in the tropics was due to a more stable climate when compared to temperate regions, stating that ‘the conditions of existence are most favourable’ there (Wallace 1891, 49).

There have been even fewer systematic tests of whether more gaudy coloration is found at lower latitudes, but Cooney et al. (2022) found that passerine birds across the globe are generally more colourful in the tropics compared to temperate regions, and suggested that this was due to greater intensity of sexual selection in tropical male birds. Possibly, this could be driven by increased selection for distinguishable visual signals in more diverse, ‘noisy’ tropical habitats (see Adams et al. 2014; Dalrymple et al. 2018). On the other hand, Dalrymple et al. (2015) found that species of birds, butterflies and flowers along the west coast of Australia were more diverse and more saturated in colour if found *further* from the equator suggesting perhaps that greater coloration in the tropics might be due to observational bias. They suggested that the visual impact of some dazzling tropical species might be enough to dominate perceptions of tropical locations as a whole (see also, Friedman and Remeš 2017).

Gloger's rule on the one hand, and ideas put forward by the 19th‐century naturalists on the other generate opposing predictions. The former suggests that homeotherms will be darker in the tropics; the latter that they will be more colourful. We decided to test these opposing ideas in terrestrial mammals, one of the two classes of endotherms, both across the class and within certain orders. Mammals have, historically, received less attention in regard to coloration compared to birds and invertebrates, in part owing to a consensus that mammals use coloration for little other than background matching (Caro and Koneru 2021). Since the 19th‐century naturalists' ideas pertained primarily to bird and insect species (to which Gloger's rule does not pertain), it remains unknown whether their conjectures apply widely to mammals.

The research that has been conducted on mammals has either been on intraspecific differences (Lai et al. 2008; Newell et al. 2021) or comparative analyses of a single group of mammals such as the primates. For example, Kamilar and Bradley (2011) found support for Gloger's rule in primate species when examining dorsal body surfaces, finding that they are darker in habitats with increased evapotranspiration rates. More recently, Caro et al. (2021) found that primate pelage is darker in warmer, more humid environments, particularly within cercopithecoid species. Despite this relative lack of research attention to date, mammals are actually an ideal taxon for investigating these sorts of global‐scale questions as they have a global distribution, inhabit a wide range of habitat types across the world, and are also subject to differing ecological pressures faced by different taxonomic groups. Luckily, there is a wealth of existing open‐source databases containing information on their social, ecological and geographical traits, which provide ideal starting points for comparative analyses (IUCN 2019; Jones et al. 2009; Myers et al. 2014; Smithsonian National Museum of Natural History 2008).

Therefore, our class‐level analyses allowed us to look at these gradients at a global scale, broadening understanding of mammal coloration beyond the few groups commonly studied (i.e., carnivores and primates), while our order‐level analyses allowed us to explore associations in groups facing different ecological and foraging selection pressures. Further, and more specifically, we expected that, should mammals conform to the complex version of Gloger's rule, species would sport darker coloration on most of their body, particularly on their torso and legs, in warmer, more humid areas. Additionally, we also expected to observe redder coloration on all areas of the body in more arid regions. If 19th‐century speculations held, however, we thought that the body, but especially the head and tail, would be redder, more colourful, more contrasting and more patterned because these distal areas are often used in communication and can be facultatively displayed while otherwise maintaining crypsis (Stuart‐Fox et al. 2004; Kamilar and Bradley 2011; Caro, Walker, Santana, et al. 2017; Howell and Caro 2024).

## 2 Methods

### 2.1 Sample Selection

We used online databases to characterise extant mammal coloration. The pruned Upham et al. (2019) phylogeny provided a sample of all extant, wild, terrestrial, non‐volant mammals (4411 spp.). All data collected were matched to the scientific binomials of these species as written on the phylogenetic tree tip labels. All bats (order Chiroptera), manatees and dugongs (order Sirenia) and whales and dolphins (clade Cetacea within order Cetartiodactyla) were omitted owing to the different lighting conditions present underwater (Meredith et al. 2013; McGowen et al. 2014) and in the caves inhabited by many bat species (Frick et al. 2020; Straka et al. 2020). Domesticated species were removed as artificial selection has a major impact on coat coloration and can produce ecologically irrelevant colours and patterns (Cieslak et al. 2011).

### 2.2 Data Collection

#### 2.2.1 Coloration Data

Photographs of each species were chosen first from iNaturalist (2021). For species where this resource had no suitable photographs, images were sourced instead from the Global Biodiversity Information Facility (GBIF; 2021). For species where no suitable photographs were available from either database, a final image search was conducted via Nature Picture Library (2021). Search terms used to find species photographs were strictly limited to the full binomials of each species, as a measure of avoiding species misidentification. The first five photographs of a species that met these criteria were used for data collection: (1) no over‐/undersaturation of the photograph, and no filters applied to the photograph, (2) the whole animal was visible, (3) the whole animal was in focus, (4) the individual was photographed in its natural habitat (e.g., no human‐made structure took up the background) and (5) there was a range of lighting conditions present across the photographs chosen for a single species. No limitations were applied to the time period in which the photographs were taken, and all images were accessed from September 2020 to February 2022 inclusive. For species with high levels of between‐individual variation that caused difficulty in scoring the colours of the individuals in the five photographs, an additional five were used.

If a species was colour polymorphic, and this polymorphism was associated with a certain subspecies, photographs of the nominate subspecies were used; otherwise, it was the morph for which the most photographs were available. Additionally, if species were sexually dichromatic (seen in only 2.4% of the total sample), only males were scored in case there was greater selection pressure on females to remain cryptic in colour. Species were considered sexually dichromatic if the sexes were described as having different coat colours in volumes 1–8 of Handbook of the Mammals of the World (HMOW; Mittermeier et al. 2009–2018). For species with seasonal colour change, only summer morphs were used. Only photographs of adult individuals were scored, to avoid the potential for discrepancies caused by ontogenetic colour change.

To ensure that species identities had not been mislabelled in photographs, locations of where photographs were taken were checked against the corresponding species' geographic range (as mapped on the IUCN Red List website; IUCN 2019). Plate illustrations of the species in HMOW volumes gave a better understanding of the specific markings and features used to tell species apart from their congeners (Mittermeier et al. 2009–2018). The total number of species for which acceptable photographs were available was 2726 spp. (62% of species surveyed).

**FIGURE 1:**
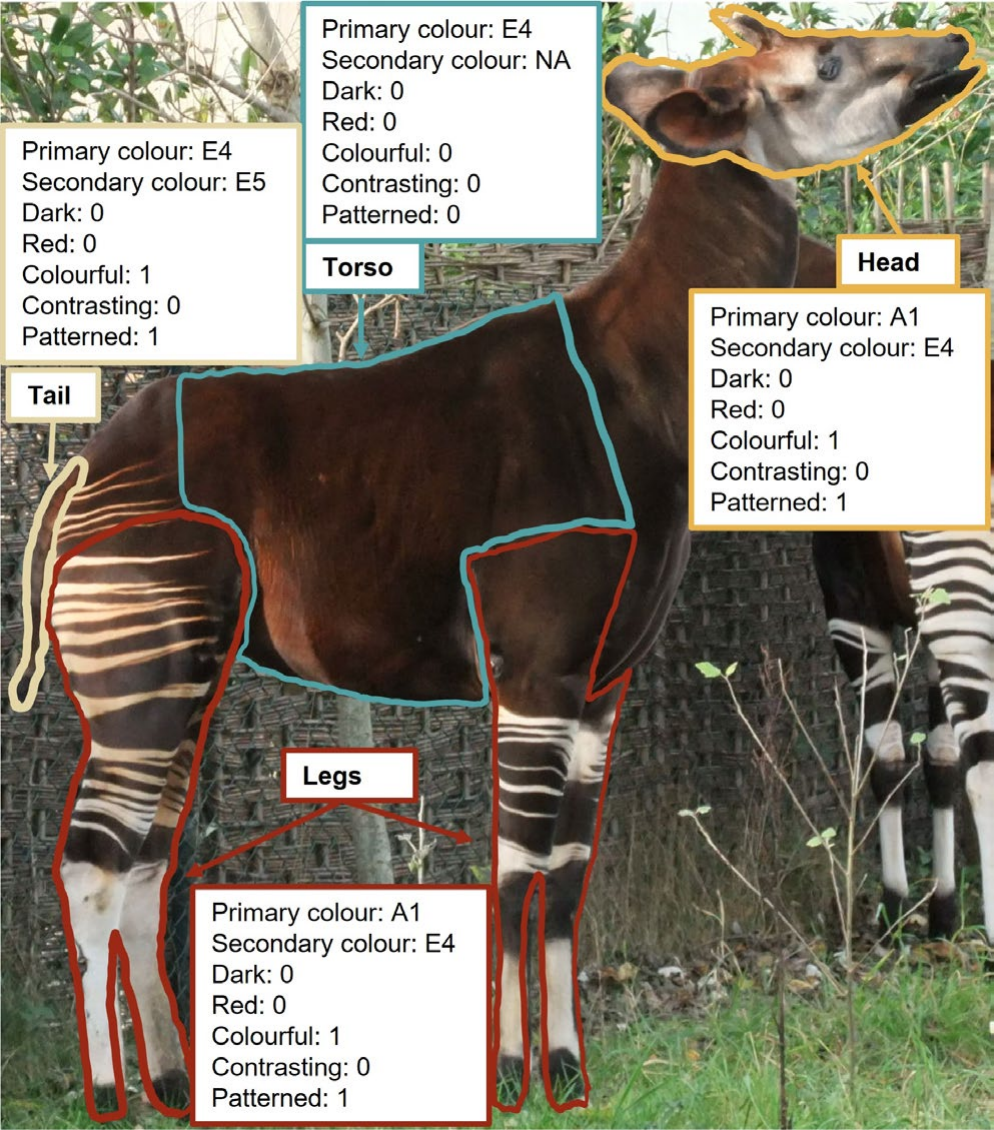
An okapi ( *Okapia johnstoni* ), with body regions highlighted and annotated to showcase the different colour measures scored in this study (for binomial 1/0 scores, 1 = present, 0 = absent). Original photograph from WikiMedia.

**FIGURE 2:**
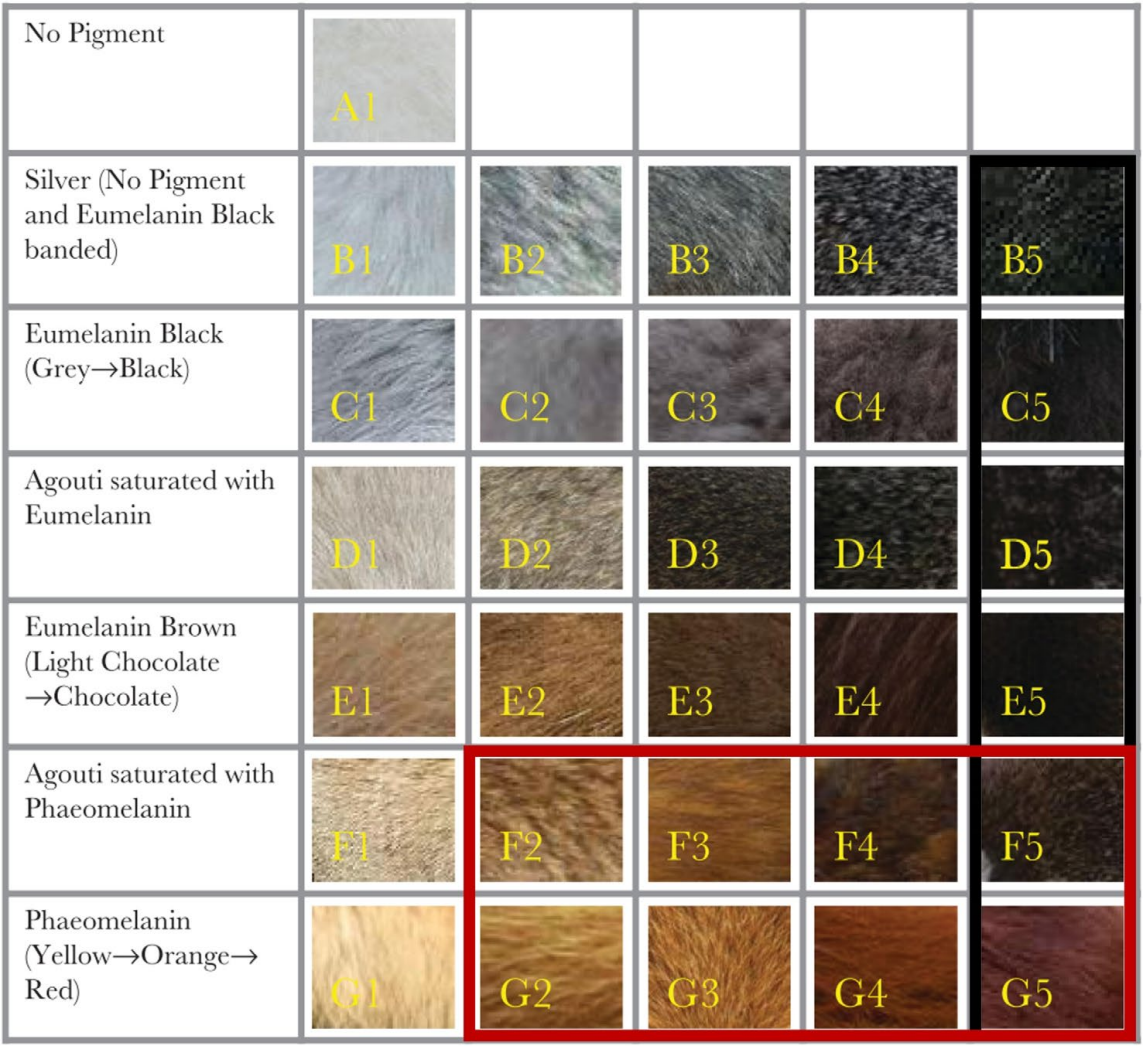
Pelage melanisation chart used to score relative presence of eumelanin and phaeomelanin in mammalian hair (Caro, Walker, Rossman, et al. 2017). Categories within the black border are all colour scores classified as ‘dark’, and those within the red border are all scores classified as ‘red’.

For each species, every colour on the head, torso, legs and tail (Figure 1) was scored, as well as the total number of distinct colours on each body region, using a pelage melanisation chart employed with success in previous studies of mammal coloration (Caro, Walker, Rossman, et al. 2017; Caro, Walker, Santana, et al. 2017; Newell et al. 2021; Figure 2). The chart categorises colour variation in mammalian hair into 31 categories, taking into account varying manifestations of eumelanin and phaeomelanin, and different levels of richness of pigmentation. This chart addresses pelage coloration specifically, and so it is important to note that mammal skin coloration was not scored in this study; moreover, mechanisms underlying skin and hair coloration are different (Bradley and Mundy 2008) and functions are likely to differ (e.g., bare skin for signalling, and hair for protective coloration). The photographs and the chart were each viewed on identical Acer V226HQL monitors that were set to the same brightness and colour specifications.

Each of the four body regions was scored for primary colour (the most prevalent colour on the body region, comprising ≥ 50% of each body part's surface as observed by eye), then secondary colour and so on until all possible colours on the head, torso, legs and tail had been scored. In cases where no single colour covered ≥ 50% of the surface, the otherwise most prevalent colour was designated the primary colour. The number of total distinct colours per body region was used as a measure of colourfulness.

To explore the various ecogeographical rules driving coloration, both red coloration and dark coloration were considered separately to other possible colours on the chart. A body region was deemed as primarily ‘*red*’ if the primary colour of that body region was assigned a value of F2–5 or G2–5 on the colour chart (see Figure 2). *Dark* coloration constituted a primary colour score of 5 (letters B through G; see Figure 2). To be conservative, colours were considered to be *contrasting* on each body region if the primary colour of a body region achieved any score of 1 on the chart, and the secondary colour any score of 5 (or vice versa; Figure 2). It is important to note that for all measures, each body region was assessed independently of other parts of the body, for example, a species with a white head and a black body would not be considered to have contrasting head coloration, whereas black‐and‐white coloration upon the head would be considered to have contrasting head fur.

**FIGURE 3:**
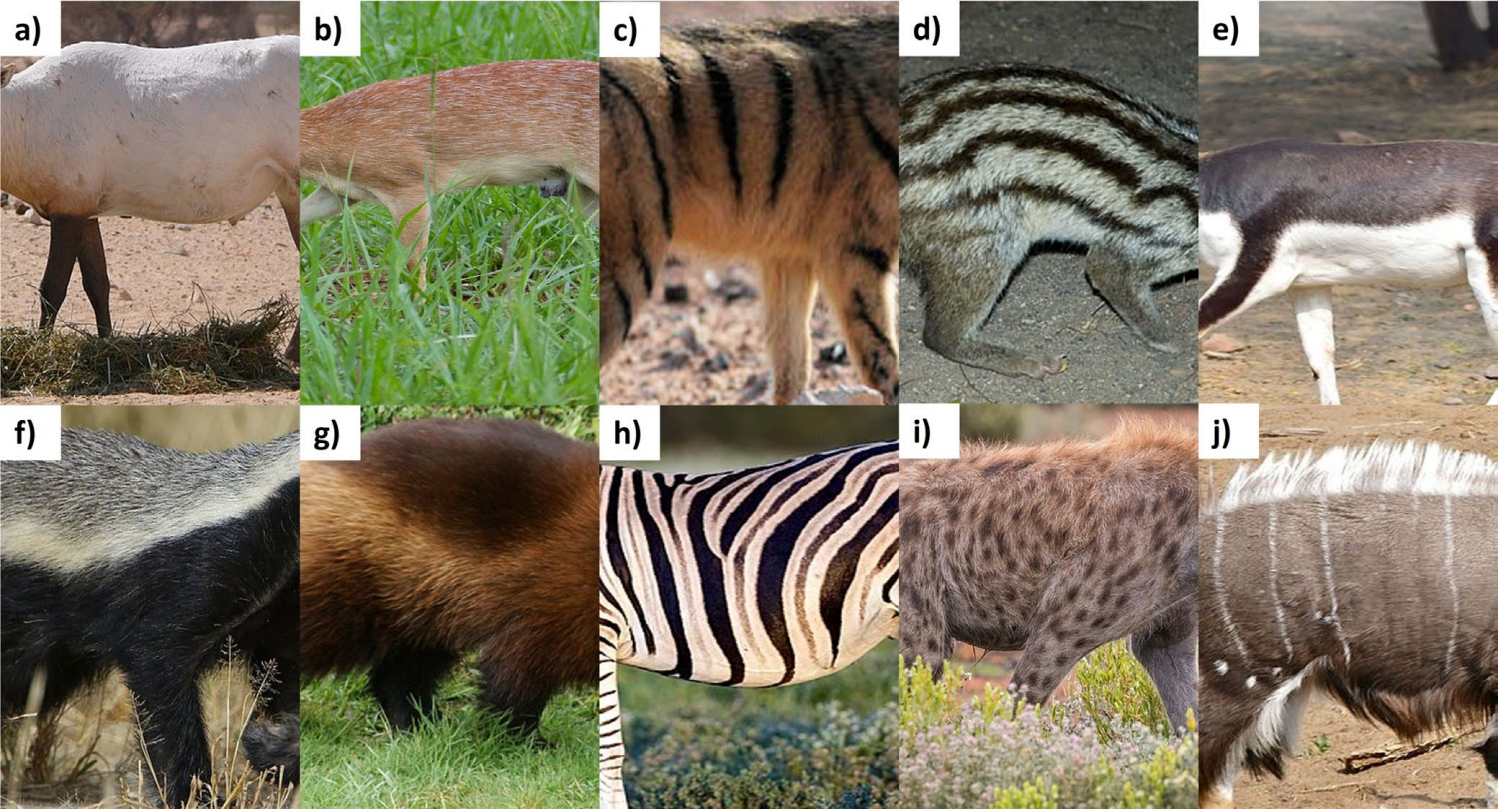
Examples of each of the 10 torso pattern scores. (a) Arabian oryx ( *Oryx leucoryx* ) with pattern 0—uniform torso coloration; (b) Sharpe's grysbok ( *Raphicerus sharpei* ) with pattern 1—flecked torso coloration; (c) Aardwolf ( *Proteles cristata* ) showcasing pattern 2—vertical torso striping; (d) Grandidier's vontsira ( *Galidictis grandidieri* ) with pattern 3—longitudinal torso stripes; (e) Blackbuck ( *Antilope cervicapra* ) showing pattern 4—countershaded torso patterning; (f) Honey badger ( *Mellivora capensis* ) with pattern 5—reverse countershading; (g) Wolverine ( *Gulo gulo* ) with pattern 6—irregular blocks of colour; (h) Plains zebra ( *Equus quagga* ) showing pattern 7—disordered striping resembling contour lines; (i) Spotted hyaena ( *Crocuta crocuta* ) with pattern 8—spotted torso patterning; (j) Nyala ( *Tragelaphus angasii* ) showing pattern 9—spots and stripes. Photographs from WikiMedia.

**FIGURE 4:**
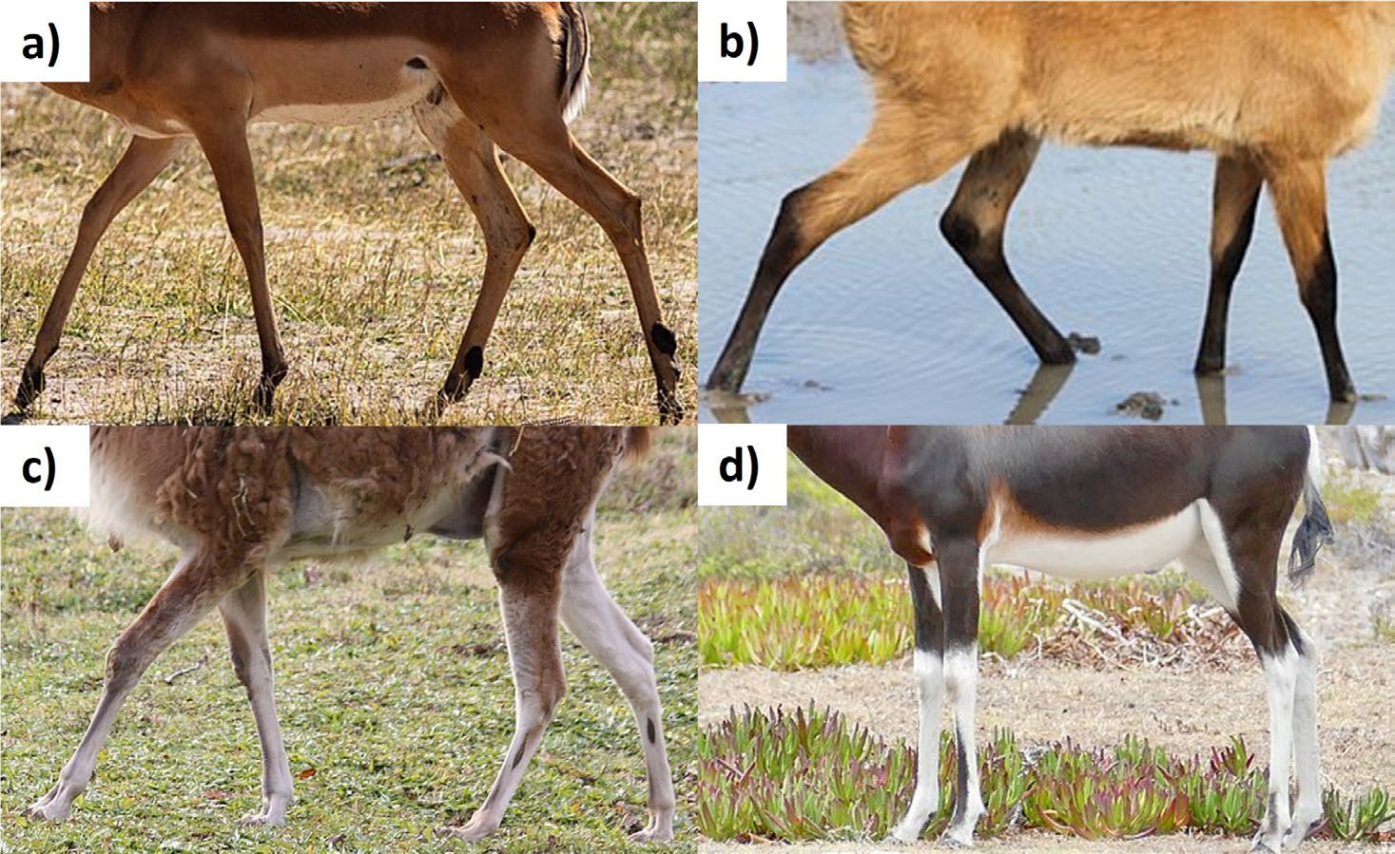
Examples of each of the four leg pattern scores. (a) Impala ( *Aepyceros melampus* ) with pattern 0—uniform leg coloration; (b) Marsh deer ( *Blastocerus dichotomus* ) with pattern 1—horizontal banding; (c) Guanaco ( *Lama guanicoe* ) showing pattern 2—vertical banding; (d) Bontebok ( *Damaliscus pygargus* ) with pattern 3—complex patterns. Photographs from WikiMedia.

Pelage patterns were described as follows. Head: 0 = uniformly‐coloured, 1 = adjacent blocks of colour, 2 = stripes, 3 = complex patterns (see fig. 5 of Howell and Caro 2024). Torso: 0 = uniformly‐coloured, 1 = flecked, 2 = vertical stripes, 3 = longitudinal stripes, 4 = countershaded (dark dorsum, light ventrum), 5 = reverse countershaded (light dorsum, dark ventrum), 6 = irregular blocks of colours, 7 = disordered stripes resembling contour lines, 8 = spots, 9 = stripes and spots (Figure 3; categories adapted from Howell et al. 2021). Legs: 0 = uniformly‐coloured, 1 = horizontal bands, 2 = vertical bands, 3 = complex patterns (Figure 4). Tails: 0 = uniformly‐coloured, 1 = longitudinal stripes, 2 = adjacent blocks, 3 = alternating bands, 4 = flecked, 5 = spotted, 6 = complex patterns (see fig. 6 of Howell and Caro 2024). The category of ‘complex patterns’ for the head, leg and tail regions was a catch‐all category for patterns that could not be scored in one of the other available categories.

Colour scores from a subset of the data comprising 10% of species from each order were evaluated for accuracy using inter‐observer reliability analyses. Agreement was found in > 80% of cases, surpassing the recommended agreement threshold of 70% (Martin and Bateson 2007; see Supporting Information for full details of this process, Table S1).

#### 2.2.2 Ecological Data

**FIGURE 5:**
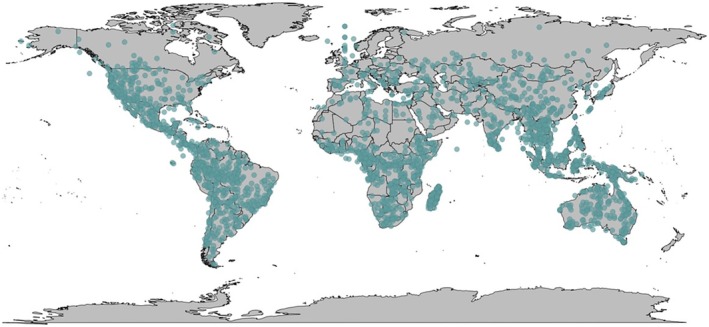
A global map of locations for all species sampled in this study, represented by the median latitudinal and longitudinal extents of each species' range. Each blue dot on the map represents a single species.

For all ecological variables used in the phylogenetic comparative models, data were obtained from PanTHERIA (Jones et al. 2009). Mean annual temperatures of each species' geographic range were recorded in degrees Celsius (°C). Mean monthly precipitation values from each species' geographic range were recorded in millimetres per month (mm/m), as were mean monthly actual evapotranspiration rate values (AET, referred to as simply ‘evapotranspiration’ in this manuscript). Mid‐range latitude and longitude (°) data, defined as the median latitudinal and longitudinal extents of each species' range, were also extracted from this database (see Figure 5 for a global map of locations of the sampled species, calculated with mid‐range data points for latitude and longitude).

### 2.3 Data Analysis

All analyses were conducted as logistic regressions in a Bayesian phylogenetic framework using the R package ‘MCMCglmm’ (Hadfield 2010). Prior to running each model, both the sample and phylogeny would be temporarily trimmed down to only the species with no missing data points from the certain order being tested within that model. The phylogeny was accessed in R via the use of package ‘phangorn’ (Schliep 2011). Models were run if the final sample was 50 species or more after trimming (Lenzner et al. 2021; Santini et al. 2016). See Supporting Information for all code used in this study.

The data from each colour variable were manipulated into binary format prior to construction of the models, necessary for logistic regressions. For example, when investigating red coloration on the torso, mammal species which were considered primarily ‘red’ were re‐scored as 1, otherwise 0. When looking at pattern, categories were simplified into patterned (1) and un‐patterned or uniform (0) for each body region. For example, head patterns 1–3 were all re‐scored as 1, whereas scores of 0 remained the same. All predictor variables in this study were comprised of continuous data; these variables were scaled to a mean of 0 and a variance of 1 prior to their input into the models, as is standard for this model procedure (Sheard et al. 2020). This allowed for improved interpretability of model coefficients.

Priors for the fixed effects of these regressions were assigned to each model using MCMCglmm's ‘gelman.prior’ command. The residual variance was fixed at 1 and the phylogenetic variance was set to an improper prior (*V* = 10–10, *v* = −1) in all models, as is standard for this particular method (Hadfield 2010). Using the current accepted mammal phylogeny (Upham et al. 2019), 100 tree topologies were randomly selected prior to running each model. These were used as random effects. A dummy run on an arbitrary tree preceded each model, in order to determine the start point. These dummy runs ran for 11,000 iterations, with a burn‐in of 1000 and a thin interval of 10. The models were then run across each of the 100 tree topologies for 11,000 iterations, with a burn‐in of 1000 and a thin interval of 1000, providing a total posterior sample of 1000 (10 per tree). Convergence of model parameters was checked via the Gelman‐Rubin statistic (the potential scale reduction (PSR) factor among chains should be < 1.1) (Gelman and Rubin 1992); all PSR factors met this criterion.

Variance inflation factors (VIFs) of the predictor variables were calculated for each model to check for potential multicollinearity between variables. All VIF values for mid‐range latitude and for temperature were below the suggested cut‐off point of 10, allowing for independent interpretation of latitude and temperature results in each of the models (O'Brien 2007). However, VIF values for precipitation and evapotranspiration were far above the threshold of 10 in models focused on orders Carnivora, Cetartiodactyla, Dasyuromorphia, Diprotodontia and Rodentia. This correlation between predictor variables invalidated the findings of these models. Therefore, a principal component analysis (PCA) was performed on the precipitation and evapotranspiration data using the ‘tidyverse’ collection of R packages (Wickham and Grolemund 2017). The resulting principal component (PC) score of ‘PC1’, which was found to explain 94.3% of the total variance in those data, was put into a secondary round of models to investigate any effects that mean precipitation and evapotranspiration rates might have on the evolution of coloration and patterns in those orders with high collinearity. Overall, our analyses were comprised of 64 models focusing on Gloger's rule, and 91 models investigating various aspects of the 19th‐century naturalists' conjectures about bright coloration.

## 3 Results

### 3.1 Gloger's Rule

#### 3.1.1 General

Should mammals conform to Gloger's rule, we expected to find that species would sport darker coloration on most of their body, particularly on the torso and legs, in warmer, more humid regions, to match their backgrounds. Many of our comparative tests confirmed this aspect of Gloger's rule. We also expected redder coloration on these areas of the body in more arid regions, if mammals were to follow both aspects of the complex definition of the rule. However, our comparative tests did not confirm this facet of Gloger's rule, with the single significant result for redness suggesting the opposite.

#### 3.1.2 Darker Coloration

**FIGURE 6:**
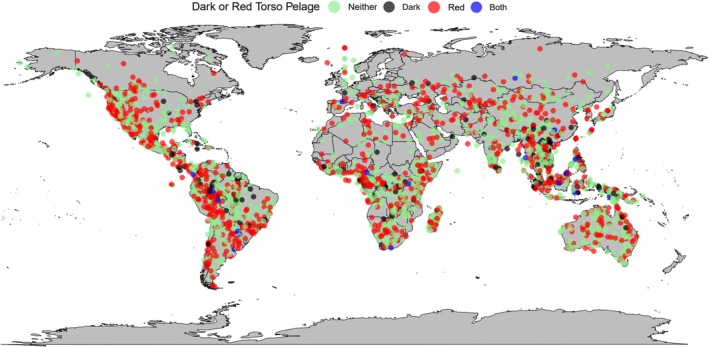
A global map of the main mammal torso coloration types tested in this study, dark fur coloration and red fur coloration. Each coloured dot represents a species: Black = dark‐furred species (colour codes B‐E5; Figure 2), red = red‐furred species (colour codes F and G2–4; Figure 2), blue = species with dark and red fur (colour codes F5 and G5; Figure 2) and green = species with neither dark nor red fur (all remaining colour codes; Figure 2).

**FIGURE 7:**
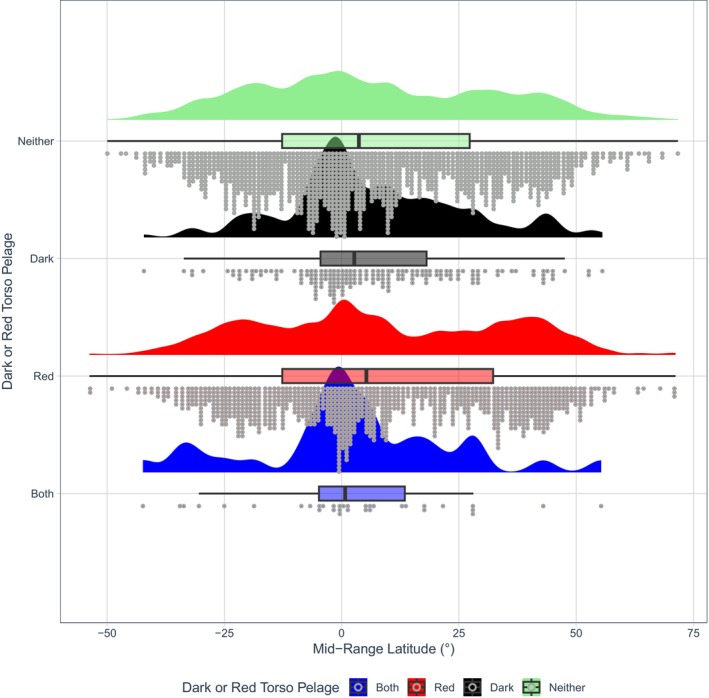
A raincloud plot showing dark and/or red torso pelage coloration against latitude. Half‐density violin plots represent sample distribution, with each dot representing a single species within the sample. Mammals as a whole were more likely to evolve darker torso coloration (colour codes B‐E5; Figure 2) at lower latitudes (*β* = −0.4190, *p* = 0.002, Table S2).

Darker torsos were more likely to evolve in species living *closer* to the equator when tested at the class level (*β* = −0.4190, *p* = 0.002, Table S2, Figures 6 and 7). This result was also found for the Australian herbivorous marsupials of Diprotodontia (*β* = −2.3402, *p* = 0.012, Table S3) and rodents (*β* = −0.5511, *p* = 0.006, Table S4). In addition, rodents were more likely to evolve darker torso coloration in warmer areas (*β* = 1.0928, *p* = 0.024, Table S4).

**FIGURE 8:**
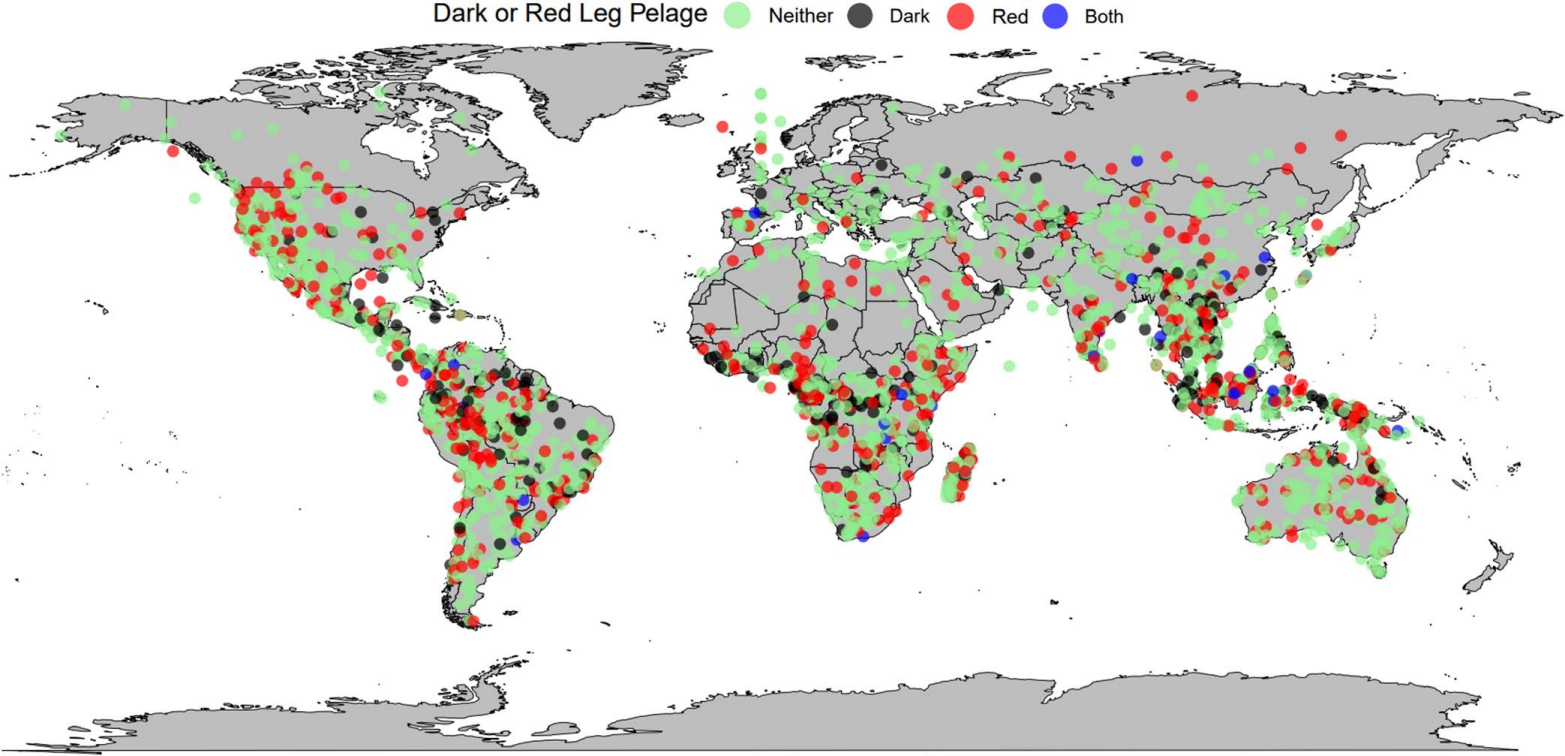
A global map of the main mammal leg coloration types tested in this study, dark fur coloration and red fur coloration. Each coloured dot represents a species: Black = dark‐furred species (colour codes B‐E5; Figure 2), red = red‐furred species (colour codes F and G2–4; Figure 2), blue = species with dark and red fur (colour codes F5 and G5; Figure 2) and green = species with neither dark nor red fur (all remaining colour codes; Figure 2).

**FIGURE 9:**
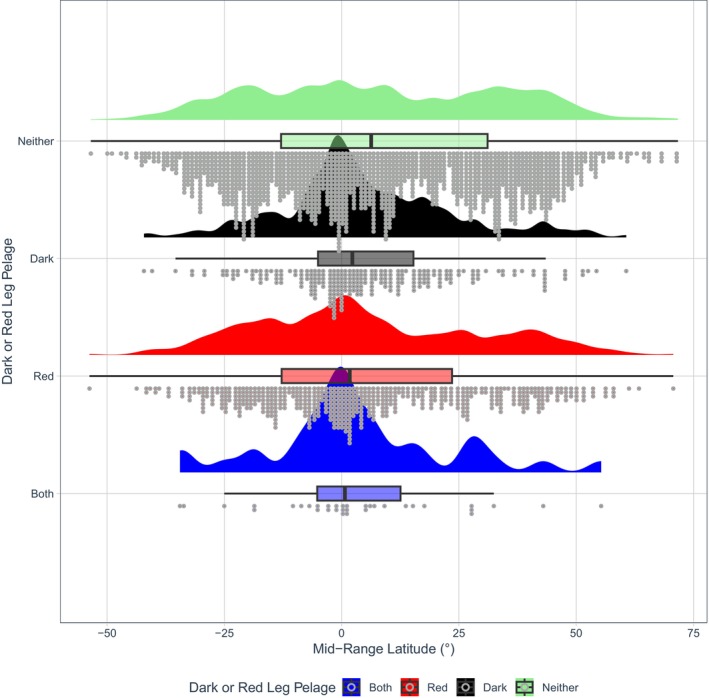
A raincloud plot showing dark and/or red leg pelage coloration against latitude. Half‐density violin plots represent sample distribution, with each dot representing a single species within the sample. Mammals as a whole were more likely to evolve darker leg coloration (colour codes B‐E5; Figure 2) at lower latitudes (*β* = −0.4939, *p* < 0.001, Table S5).

Across mammals, darker legs were also more likely to evolve in species living *closer* to the equator (*β* = −0.4939, *p* < 0.001, Table S5, Figures 8 and 9). Similarly, this association was replicated for Diprotodontia (*β* = −2.5937, *p* = 0.004, Table S6) and Rodentia (*β* = −0.7344, *p* < 0.001, Table S7).

**FIGURE 10:**
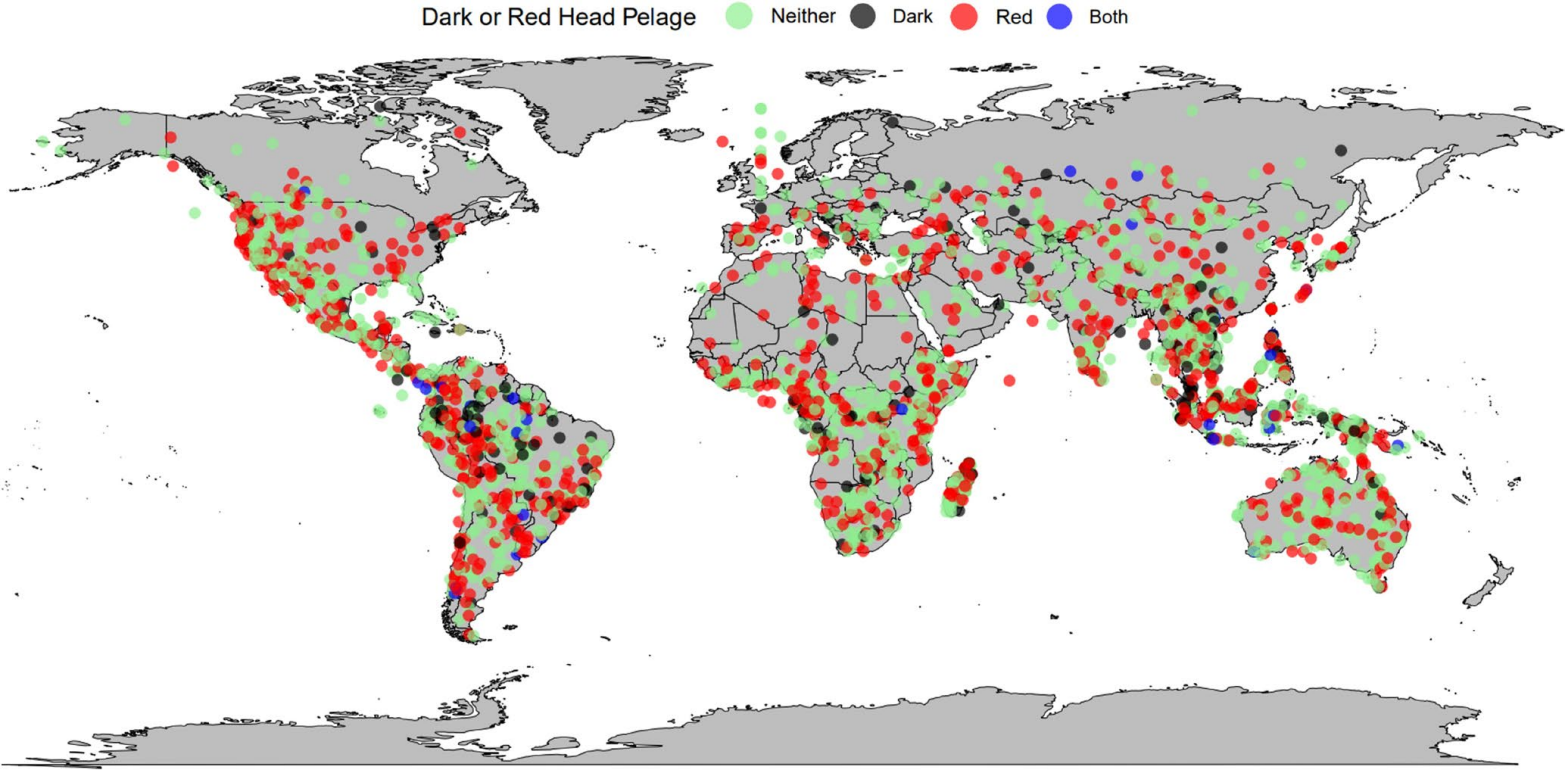
A global map of the main mammal head coloration types tested in this study, dark fur coloration and red fur coloration. Each coloured dot represents a species: Black = dark‐furred species (colour codes B‐E5; Figure 2), red = red‐furred species (colour codes F and G2–4; Figure 2), blue = species with dark and red fur (colour codes F5 and G5; Figure 2) and green = species with neither dark nor red fur (all remaining colour codes; Figure 2).

**FIGURE 11:**
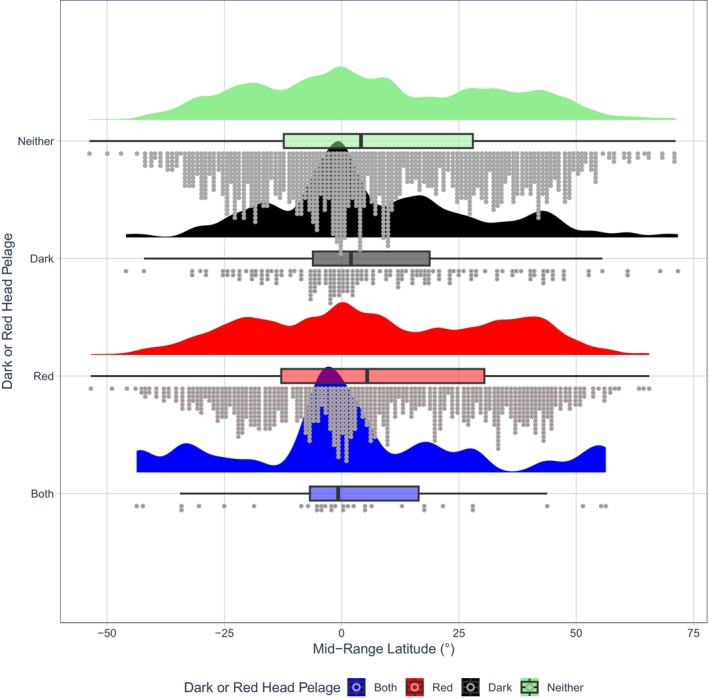
A raincloud plot showing dark and/or red head pelage coloration against latitude. Half‐density violin plots represent sample distribution, with each dot representing a single species within the sample. Mammals as a whole were more likely to evolve darker head coloration (colour codes B‐E5; Figure 2) at lower latitudes (*β* = −0.3677, *p* = 0.008, Table S8).

**FIGURE 12:**
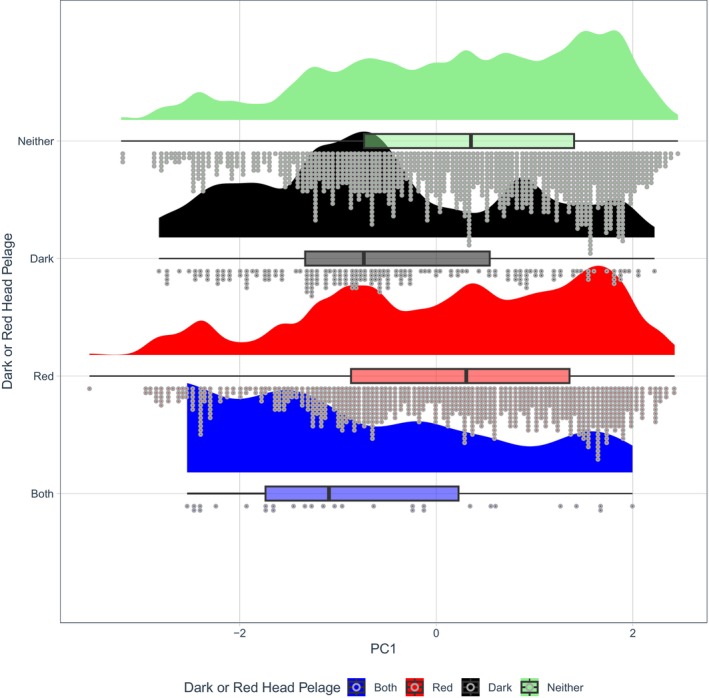
A raincloud plot showing dark and/or red head pelage coloration against joint mean precipitation and mean evapotranspiration values (mm/m; PC1). Half‐density violin plots represent sample distribution, with each dot representing a single species within the sample. Mammals as a whole were more likely to evolve darker head coloration (colour codes B‐E5; Figure 2) in habitats with higher joint precipitation and evapotranspiration rates (*β* = 0.3264, *p* = 0.016, Table S8).

Darker head pelage was more likely to evolve in species living *closer* to the equator, again both across the class (*β* = −0.3677, *p* = 0.008, Table S8, Figures 10 and 11) and among rodents (*β* = −0.6074, *p* = 0.002, Table S9). In addition, darker head coloration was associated with a joint *increase* in precipitation and evapotranspiration rates both across mammals (*β* = 0.3264, *p* = 0.016, Table S8, Figure 12) and rodents (*β* = 0.4961, *p* = 0.044, Table S9; a ‘joint’ increase refers to the predictor variable of PC1 generated from the PCA constructed from the variables of precipitation and evapotranspiration rates).

**FIGURE 13:**
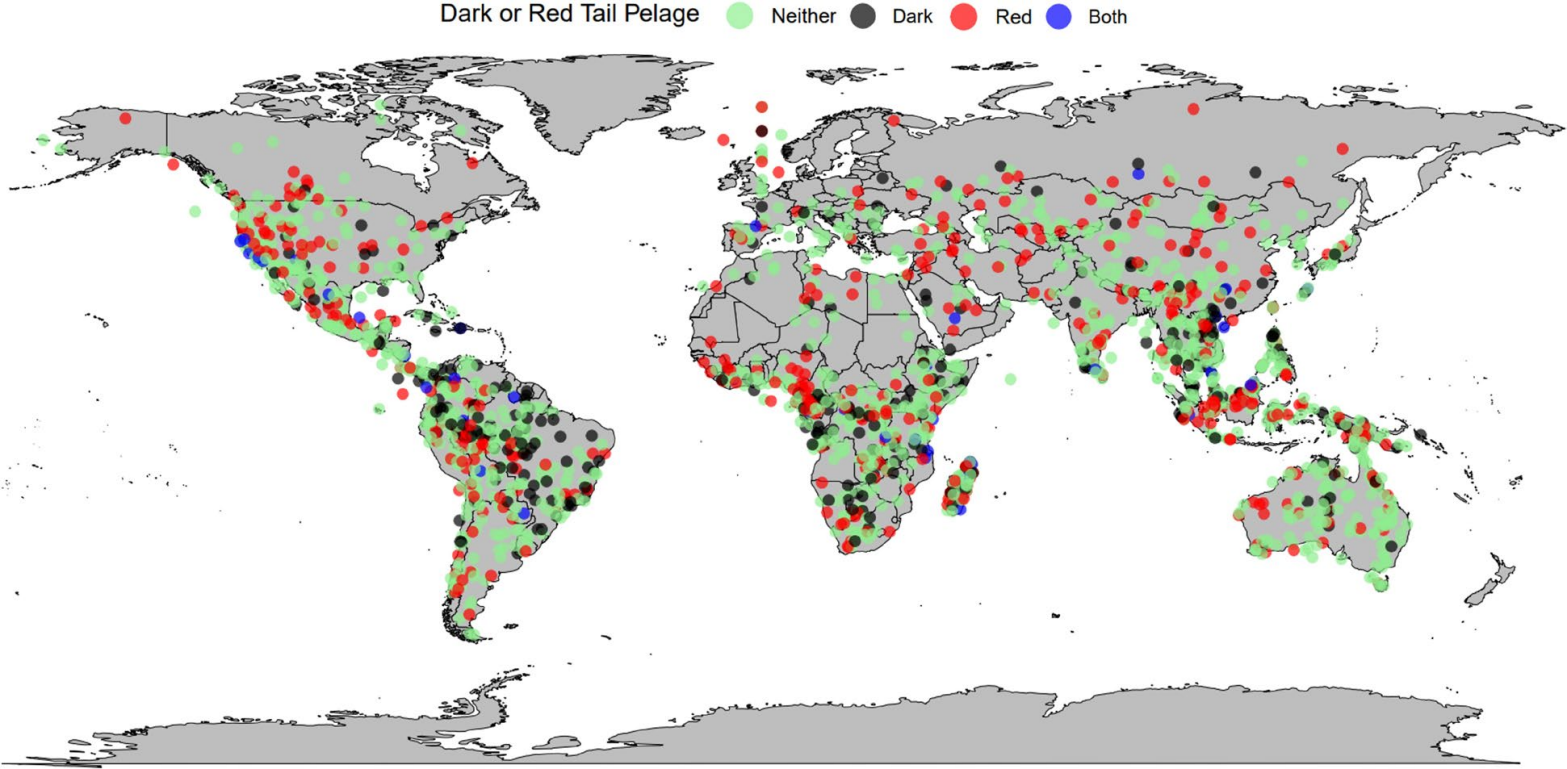
A global map of the main mammal tail coloration types tested in this study, dark fur coloration and red fur coloration. Each coloured dot represents a species: Black = dark‐furred species (colour codes B‐E5; Figure 2), red = red‐furred species (colour codes F and G2–4; Figure 2), blue = species with dark and red fur (colour codes F5 and G5; Figure 2) and green = species with neither dark nor red fur (all remaining colour codes; Figure 2).

**FIGURE 14:**
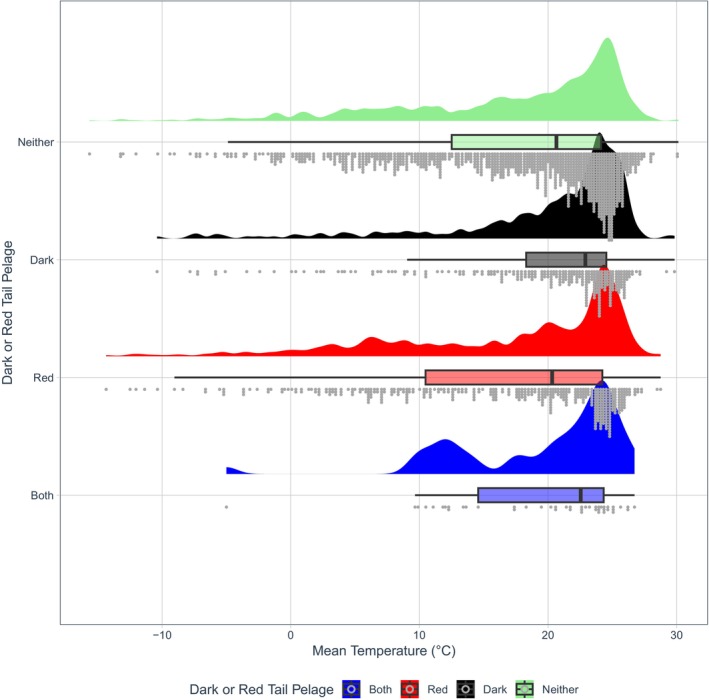
A raincloud plot showing dark and/or red tail pelage coloration against mean temperature. Half‐density violin plots represent sample distribution, with each dot representing a single species within the sample. Mammals as a whole were more likely to evolve darker tail coloration (colour codes B‐E5; Figure 2) at higher temperatures (*β* = 0.2762, *p* = 0.028, Table S11).

Of all orders analysed, artiodactyls were the only taxon likely to evolve darker tail coloration when *closer* to the equator (*β* = −0.8731, *p* = 0.040, Table S11). Across mammals, darker‐coloured tails were observed in warmer areas (*β* = 0.2762, *p* = 0.028, Table S10, Figures 13 and 14).

#### 3.1.3 Redder Coloration

Contrary to predictions, primates were more likely to evolve redder tail coloration in areas with *higher* precipitation rates, not lower (*β* = 0.9421, *p* = 0.022, Table S12). No other significant findings were found for redder coloration when matched to the independent variables (see Supporting Information).

### 3.2 Historical Conjectures: Colour and Contrast

#### 3.2.1 General

If the 19th‐century naturalists' observations around latitudinal gradients of coloration held true, we would expect mammals inhabiting tropical environments to sport more reddish coloration across body regions, be more colourful and have more contrasting colours than their temperate counterparts. We found very little support for any of these conjectures in this study.

#### 3.2.2 Redder Coloration

As described above, primates were more likely to evolve primarily red tail coloration in areas with *higher* precipitation rates (*β* = 0.9421, *p* = 0.022, Table S12).

#### 3.2.3 Colourful Pelage

Primates were more likely to evolve colourful torso pelage (i.e., a torso comprising a greater number of distinct colours) when inhabiting environments *closer* to the equator (*β* = −0.7765, *p* = 0.010, Table S13). Rodents were more likely to evolve such torso coloration in warmer regions (*β* = 0.5052, *p* = 0.032, Table S14) and carnivorous marsupials of Australia (order Dasyuromorphia) were likely to evolve this multicoloured torso pelage when precipitation and evapotranspiration rates jointly *increased* (*β* = 2.2237, *p* = 0.020, Table S15) as is characteristic of the tropics. Rodents, on the other hand, were more likely to evolve such torso coloration when *further* from the equator (*β* = 0.8295, *p* < 0.001, Table S14).

Contrary to predictions, mammals were more likely to evolve more colourful head pelage when living *further* from the equator (*β* = 0.2867, *p* = 0.018, Table S16), a result replicated in rodents (*β* = 0.4779, *p* = 0.018, Table S17).

Of all the orders analysed, primates were the only one in which more colourful tails were seen in warmer areas (*β* = 0.6475, *p* = 0.034, Table S18).

#### 3.2.4 Contrasting Coloration

Again, against 19th‐century speculations, contrasting torso coloration was more likely to evolve in mammals living *further* from the equator when investigated across the entire class (*β* = 0.2640, *p* = 0.006, Table S19), a result replicated in Dasyuromorphia (*β* = 3.0051, *p* = 0.006, Table S20).

And again, contrasting leg coloration was more likely to be seen in mammals that lived *further* from the equator (*β* = 0.3604, *p* = 0.002, Table S21), particularlyor rodents (*β* = 0.4767, *p* = 0.020, Table S22).

In rodents, contrasting head pelage was more likely to be found in areas with *higher* precipitation and evapotranspiration rates (*β* = 0.2423, *p* = 0.026, Table S23).

### 3.3 Distinct Patterning

#### 3.3.1 Patterned Pelage

In mammals, patterned torsos (i.e., stripes, spots, adjacent blocks of coloration and so on as shown in Figure 3) were more likely found in areas with *higher* precipitation and evapotranspiration rates (*β* = 0.1721, *p* = 0.048, Table S26). In primates, torso patterning was more likely to be seen in species living *closer* to the equator (*β* = −0.6973, *p* = 0.018, Table S24). On the other hand, rodents were more likely to evolve torso patterning living *further* from the equator (*β* = 0.6591, *p* < 0.001, Table S25).

Against predictions, head patterning was more likely to evolve in mammals that lived *further* from the equator (*β* = 0.2871, *p* = 0.032, Table S27), a result replicated in rodents (*β* = 0.4686, *p* = 0.032, Table S28).

Of all orders investigated, only primates were more likely to evolve patterned tails when inhabiting warmer areas (*β* = 0.6583, *p* = 0.018, Table S29).

To conclude, it should be noted that although we found multiple lines of evidence supporting one complex aspect of Gloger's rule (eumelanin), and a little support for the 19th‐century naturalists' predictions (see Table 1 for summary of significant results), there were a great many models that returned no results of significance and will not be discussed in detail here (see Tables S30–S156 for all non‐significant results).

**TABLE 1:** Summary of significant correlations. A positive *Z*‐score indicates a positive correlation, while a negative *Z*‐score indicates that the correlation is negative.

**Tests of Gloger's rule**
*Darker Coloration*
Mammals as a whole more likely to have darker torsos at lower latitudes; *β* = −0.4190, *p* = 0.002, Table S2
Diprotodontia more likely to have darker torsos at lower latitudes; *β* = −2.3402, *p* = 0.012, Table S3
Rodentia more likely to have darker torsos at lower latitudes; *β* = −0.5511, *p* = 0.006, Table S4
Rodentia more likely to have darker torsos at higher temperatures; *β* = 1.0928, *p* = 0.024, Table S4
Mammals as a whole more likely to have darker legs at lower latitudes; *β* = −0.4939, *p* < 0.001, Table S5
Diprotodontia more likely to have darker legs at lower latitudes; *β* = −2.5937, *p* = 0.004, Table S6
Rodentia more likely to have darker legs at lower latitudes; *β* = −0.7344, *p* < 0.001, Table S7
Mammals as a whole more likely to have darker heads at lower latitudes; *β* = −0.3677, *p* = 0.008, Table S8
Mammals as a whole more likely to have darker heads at higher joint precipitation and evapotranspiration rates (PC1); *β* = 0.3264, *p* = 0.016, Table S8
Rodentia more likely to have darker heads at lower latitudes; *β* = −0.6074, *p* = 0.002, Table S9
Rodentia more likely to have darker heads at higher joint precipitation and evapotranspiration rates (PC1); *β* = 0.4961, *p* = 0.044, Table S9
Artiodactyls more likely to have darker tails at lower latitudes; *β* = −0.8731, *p* = 0.040, Table S10
Mammals as a whole more likely to have darker tails at higher temperatures; *β* = 0.2762, *p* = 0.028, Table S11
**Tests of Gloger's rule and Wallace's conjecture**
*Redder Coloration*
Primates more likely to have redder tails at higher precipitation rates; *β* = 0.9421, *p* = 0.022, Table S12
**Tests of Wallace's conjecture**
*Colourfulness*
Primates more likely to have colourful torsos at lower latitudes; *β* = −0.7765, *p* = 0.010, Table S13
Rodentia more likely to have colourful torsos at higher latitudes; *β* = 0.8295, *p* < 0.001, Table S14
Rodentia more likely to have colourful torsos at higher temperatures; *β* = 0.5052, *p* = 0.032, Table S14
Dasyuromorphia more likely to have colourful torsos at higher joint precipitation and evapotranspiration rates (PC1); *β* = 2.2237, *p* = 0.020, Table S15
Mammals as a whole more likely to have colourful heads at higher latitudes; *β* = 0.2867, *p* = 0.018, Table S16
Rodentia more likely to have colourful heads at higher latitudes; *β* = 0.4779, *p* = 0.018, Table S17
Primates more likely to have colourful tails at higher temperatures; *β* = 0.6475, *p* = 0.034, Table S18
*Contrast*
Mammals as a whole more likely to have contrasting torsos at higher latitudes; *β* = 0.2640, *p* = 0.006, Table S19
Dasyuromorphia more likely to have contrasting torsos at higher latitudes; *β* = 3.0051, *p* = 0.006, Table S20
Mammals as a whole more likely to have contrasting legs at higher latitudes; *β* = 0.3604, *p* = 0.002, Table S21
Rodentia more likely to have contrasting legs at higher latitudes; *β* = 0.4767, *p* = 0.020, Table S22
Rodentia more likely to have contrasting heads at higher joint precipitation and evapotranspiration rates (PC1); *β* = 0.2423, *p* = 0.026, Table S23
*Patterning*
Primates more likely to have patterned torsos at lower latitudes; *β* = −0.6973, *p* = 0.018, Table S24
Rodentia more likely to have patterned torsos at higher latitudes; *β* = 0.6591, *p* < 0.001, Table S25
Mammals as a whole more likely to have patterned torsos at higher joint precipitation and evapotranspiration rates (PC1); *β* = 0.1721, *p* = 0.048, Table S26
Mammals as a whole more likely to have patterned heads at higher latitudes; *β* = 0.2871, *p* = 0.032, Table S27
Rodentia more likely to have patterned heads at higher latitudes; *β* = 0.4686, *p* = 0.032, Table S28
Primates more likely to have patterned tails at higher temperatures; *β* = 0.6583, *p* = 0.018, Table S29

## 4 Discussion

### 4.1 Ecogeographical Rules and Gradients of Coloration

In a nutshell, our analyses generated strong support for terrestrial mammals adhering to the darker coloration aspect only of Gloger's rule at a macroevolutionary scale, and this extended to some order‐level findings. However, there was scant support for redder coloration being associated with warmer or more arid climates. We also found very little support for red coloration being more prevalent in the tropics as predicted by 19th‐century naturalists, and the results for multicoloured, contrasting and patterned pelage revealed findings mostly contrary to historical conjectures, at least in this taxon.

Our aim was to investigate whether mammals as a whole, along with various orders, adhered to ecogeographical rules of colour. We decided to test these ideas in terrestrial mammals to fill a gap in research, as a lot of previous research on Gloger's rule has focused on groups of birds (Burtt and Ichida 2004; Marcondes et al. 2021; Roulin and Randin 2015), and to our knowledge, no systematic investigation has explored whether mammals are more brightly coloured in the tropics.

Our class‐level analyses allowed us to look at these associations at a global scale, while our order‐level analyses allowed us to explore associations in groups facing different ecological and foraging selection pressures. The former approach enables generalisations to be made across a range of different mammal clades using a robust sample size, whereas the latter allows more nuanced associations to be teased out, taking into account order‐specific lifestyles, ecologies and evolutionary histories.

### 4.2 Gloger's Rule and Darker Pelage

The complex definition of Gloger's rule predicts that both within and across species, endotherms will evolve darker eumelanic coloration in warmer, more humid environments, and conversely, lighter and redder phaeomelanic coloration in hotter, more arid environments (Delhey 2019). Predictions regarding temperature are challenging because, according to the complex version of Gloger's rule, we would expect both darker and redder coloration in warmer regions (Delhey 2019). Of the 64 models constructed to test both aspects of this definition, a total of 10 returned significant findings in support of the rule. All were in support of the darker coloration aspect of the rule only.

We found that darker torso and leg pelage is more likely to evolve in terrestrial mammal species living closer to the equator, especially in Rodentia and Diprotodontia. In rodents, these findings have been seen by others at the intraspecific level. For instance, research conducted on wild populations of Cairo spiny mice ( *Acomys cahirinus* ) found that populations on the humid, closed habitat slope of a canyon evolve darker, more intensely eumelanic coloration than populations of the same species inhabiting the arid, open habitat slope on the opposing side of the same canyon (Singaravelan et al. 2010). Those researchers also discovered that populations of *A. cahirinus* endemic to the arid, open habitat were lighter and more intensely phaeomelanic in colour; a finding that was not extended comparatively across rodents here.

At the class level and again replicated within rodents, darker head coloration was associated with both lower latitudes and higher values in the PC1 variable of joint precipitation and evapotranspiration. This provides a clear link to the higher humidity aspect of Gloger's rule driving darker coloration. Associations for torso, leg and head regions were found at the class level and for rodents—an order comprised of a great number of small, vulnerable prey species. These results, therefore, lend indirect support to the idea that Gloger's rule might be driven by the need for efficient background matching in darker environments (see Carballo et al. 2020; Miller et al. 2019).

### 4.3 Gloger's Rule and Redder Pelage

We found no support for redder pelage being found in warmer or more arid environments, despite others' intraspecific findings (e.g., *A. cahirinus* ; Singaravelan et al. 2010) and jaguarundi (*Herpailurus yagouaroundi*; da Silva et al. 2016). It is possible that to more conclusively test this complex version of Gloger's rule, an expanded definition of ‘redness’ in colour is needed. Our marker for redness was decided by whether mammal coat colours matched with scores F and G2–5 on the colour chart used to score coloration. However, scores F1 and G1, while not strictly ‘red’ in colour, may also constitute phaeomelanin‐based coloration. Inclusion of these more golden‐yellow phaeomelanic colours might offer a more expansive test of phaeomelanic coloration. Yellow, paler beige or sandy coloration might provide better camouflage for mammals in arid environments (Fennell et al. 2019) than redness, for example. Nonetheless, we chose a more conservative measure of redness in the interests of caution.

### 4.4 Historical Conjectures: Colour and Contrast

Of the 91 models constructed to test these ideas about bright animals being found in the tropics, only 12 returned significant findings, and a great many of these results refuted these predictions rather than supported them. The majority of the *supporting* results were found within primates, which have redder tails in areas with higher precipitation, multicoloured torso pelage *closer* to the equator, and multicoloured tail pelage in higher temperatures. There was also some support found across other orders, namely rodents exhibiting colourful torsos at higher environmental temperatures and exhibiting contrasting head pelage in areas with higher precipitation and evapotranspiration rates, and Dasyuromorphia species evolving multicoloured torso pelage in areas with higher precipitation and evapotranspiration rates. The other seven significant associations all refuted the conjectures. For instance, mammals were more likely to evolve colourful head pelage when living further from the equator, and both contrasting torso and leg coloration were more likely to evolve in mammal species living further from the equator.

The idea might still hold true in other scenarios, however—perhaps ‘crimson’ coloration is poorly represented by phaeomelanic hair in mammals, but instead refers to the striking red coloration that can be found in invertebrate groups such as butterflies (Adams et al. 2014), or to the bright red facial and genital skin of some primate species (Setchell et al. 2006; Dubuc et al. 2009), or the flamboyant colours of some birds (Delhey et al. 2023). For mammalian pelage, however, these ideas do not stand up to scrutiny.

### 4.5 Historical Conjectures: Distinct Patterning

Wallace (1891) also advanced the idea that species with more distinctive patterning were more likely to be found in the tropics. Of the 32 tests conducted here to examine this proposal, only six returned significant findings, and only three were in the predicted direction. Across mammals, patterned torso coloration was associated with higher precipitation and evapotranspiration rates. In primates, species were more likely to evolve patterned torso pelage when situated closer to the equator and were more likely to evolve patterned tail coloration in areas of higher environmental temperatures. In short, extravagant patterning in the tropical habitats was largely unsupported by our data on mammals.

One reason for the lack of support for these 19th‐century conjectures could be observational bias on their part. Dalrymple et al. (2015) proposed that the diversity of cryptically‐coloured species in the tropics might simply be less memorable to humans than less diverse dazzling tropical species, therefore colourful species might dominate human impressions of tropical colours despite not being as widespread. Indeed, Wallace himself remarked that ‘for every group of brightly coloured tropical birds, there is another of equal extent whose limits are plain and sober, so that it is doubtful, whether, in proportion to the whole, more gay coloured birds are found in the tropics than in the temperate regions’ (Wallace 1869). Nonetheless, he remained fascinated with the examples of dazzling colours and patterns in tropical nature, and often favoured the argument of tropical species being more colourful (Wallace 1867, 1891). Taken in the round, we believe that the 19th‐century naturalists' conjectural ideas about bright coloration of fauna inhabiting the tropics do not apply to mammals and whether they apply to birds and insects is an open question. If they do apply to these groups, it raises questions as to whether sexual selection, aposematism, or colour vision are more common there and why (Wiens and Emberts 2025). Or, alternatively, whether the greater species diversity in the tropics (Marchese 2015) is the evolutionary cause of colour diversity there. Our analyses here show definitively that these questions are not relevant to mammals.

### 4.6 Limitations of the Study

We used photographs of mammals taken from the web and subjective matching of colours to colour charts in this study, which have been widely used in previous mammal coloration studies and have served as valuable sources of data (Caro, Walker, Rossman, et al. 2017; Caro, Walker, Santana, et al. 2017; Newell et al. 2021). These methods are tailored to collate large datasets where spectrophotometry of mammalian museum skins would be costly, subject to age‐related fading, and might involve environmentally questionable travel. Although this method may involve human classification bias and effects of background luminance, only one person was involved in scoring the coloration of over 2500 species to ensure consistency, and interobserver reliability of a subsample of these data was > 80% (Table S1). Moreover, these potential problems would not apply to our measures of patterning. For colour contrast, we took a conservative measure of the pelage extremes shown by the mammalian colour chart to decide whether a species had contrasting coloration on regions of its body. However, it should be noted that our attempt to compare predictions from Gloger's rule and 19th‐century naturalists' conjectures only considered mammal species and did not examine birds, where results could differ; certainly, they are a class exhibiting more and a greater variety of vivid colours.

A limitation of the analyses presented in this study was in the binary approach of analyses, rather than the use of multiple character states. The logistic regressions showcased the broad impacts of climatic variables on the evolution of contrasting versus non‐contrasting colours, as well as patterned versus uniformly‐coloured pelage, and so on. However, we cannot ascertain from these results whether the evolution of certain coat patterns, for example, was influenced by these climate predictors more than others.

### 4.7 Concluding Remarks

Overall, this study found overwhelming support for one aspect of the complex version of Gloger's rule (Rensch 1929) acting as a driver of coloration in mammals at a global scale, with every region of the body but the tail darker in habitats nearer the equator. It is possible that Gloger's rule is exemplified so well by mammals because of intense selection pressure for crypsis (Caro 2005). The majority of mammals are small, undefended and vulnerable to predation from a variety of predatory species including snakes, raptors and mammalian carnivores, and dark coloration that matches the dark, shady background of a closed tropical habitat is a simple way to help avoid detection. As such, our analyses hint at camouflage being a driving force behind mammals' apparent adherence to the dark coloration aspect of Gloger's rule.

## Author Contributions

**Tim Caro:** conceptualization (equal), funding acquisition (lead), investigation (supporting), methodology (supporting), resources (lead), supervision (lead), validation (equal), visualization (equal), writing – original draft (supporting), writing – review and editing (equal). **Natasha Howell:** conceptualization (equal), data curation (lead), formal analysis (lead), investigation (lead), methodology (lead), project administration (lead), software (lead), validation (equal), visualization (equal), writing – original draft (lead), writing – review and editing (equal).

## Conflicts of Interest

The authors declare no conflicts of interest.

## Supporting information


**Data S1:** ece371855‐sup‐0001‐supinfo.docx.

## Data Availability

All necessary data and code to reproduce this study can be accessed within the following Dryad data repository: https://doi.org/10.5061/dryad.2bvq83c04.
