# Supplementary material for: Gloger's Rule or Historical Conjecture? Tests in Mammals
Source: Ecol Evol. 2025 Jul 25;15(7):e71855. doi: 10.1002/ece3.71855 (PMC12291604; doi:10.1002/ece3.71855)
Supplement: Supplementary file 1 — Data S1: ece371855‐sup‐0001‐supinfo.docx. [file ECE3-15-e71855-s001.docx]

**Inter-Observer Reliability**

A randomly-generated subset of the data was collated to measure inter-observer reliability. This subset comprised 10% of the species within each Order that had photographs for colour scoring, with a stipulation that Orders containing fewer than 10 species were represented by a single species. This led to a final subset sample of 278 species. The subset was collated randomly by assigning each species in the dataset a numerical ID and using an online random number generator to select the ID numbers of the species to be included.

The subset was divided among a group of 11 volunteer observers who were unaware of the study’s aims and hypotheses. Each observer was given training in how to score the primary fur colours of each of five body regions (head, torso, rump, legs, and tail) using five example species that were not present in the subset sample. These five examples were also randomly generated via the same method, and remained the same for each observer’s training session.

For each of the species they had been randomly assigned, the volunteer observers produced their own sets of scores for the primary colours of the five body regions. These scores were decided upon independently of each-other and without any input from the primary observer (NH).

Each of the observers viewed the same photographs that NH had viewed for each species and the colour chart under the same viewing conditions. Once all observers had collated their colour scores, Index of Concordance calculations were performed using R (v.4.2.1). These compared the total number of agreements (*A*) and disagreements (*D*) between the primary observer and the volunteer observers via the equation *A* / (*A*+*D*) (Martin & Bateson, 2007). The agreement score between the primary observer and the volunteer observers was then determined as a percentage value. All but one of the calculations returned a percentage agreement above the recommended agreement threshold of 70% (see Martin & Bateson, 2007; Table S1). For the Order that did not meet the agreement threshold, Lagomorpha, the colour data collected were considered invalid and the planned models were not constructed.

**Table S1.** Summary of mean interobserver agreement scores, both at the Class level and for the Orders tested in this study (%).

| **Taxa Observed** | **Body Region** | **Total Number of Observations made by 11 Observers** | **Agreement with NH (% ± SD)** |
| --- | --- | --- | --- |
| Class-wide | All | 1,390 | 86.1 ± 0.08 |
| Class-wide | Head | 278 | 85.7 ± 0.09 |
| Class-wide | Torso | 278 | 86.7 ± 0.07 |
| Class-wide | Rump | 278 | 84.3 ± 0.12 |
| Class-wide | Legs | 278 | 84.3 ± 0.11 |
| Class-wide | Tail | 278 | 89.5 ± 0.07 |
|  | | | |
| Carnivora | All | 135 | 91.3 ± 0.12 |
| Cetartiodactyla | All | 115 | 97.4 ± 0.00 |
| Dasyuromorphia | All | 35 | 88.6 ± 0.00 |
| Diprotodontia | All | 60 | 81.4 ± 0.26 |
| Eulipotyphla | All | 65 | 82.2 ± 0.14 |
| Lagomorpha | All | 35 | 68.6 ± 0.00 |
| Primates | All | 205 | 83.6 ± 0.07 |
| Rodentia | All | 545 | 88.8 ± 0.05 |

**References**

Martin, P. & Bateson, P. (2007). *Measuring Behaviour: An Introductory Guide*. Cambridge University Press.

**Code for Data Analysis and Mapping**

All necessary data and code for the analyses and mapping conducted in this study have been made available within the following Dryad data repository: <https://doi.org/10.5061/dryad.2bvq83c04> (currently private for peer review).

Temporary reviewer link: [https://datadryad.org/share/NybnzY-RL-W1P33TblOex09P5Y8j9pqvGQ5XhJDXrdo](https://datadryad.org/stash/share/NybnzY-RL-W1P33TblOex09P5Y8j9pqvGQ5XhJDXrdo).

**Supplementary Results Tables**

Significant dark pelage results

**Table S2.** Predictors of darker torso pelage across the mammal Class, calculated with a Bayesian phylogenetic mixed model (MCMCglmm) in this and subsequent models. Significant p-values (using p<0.05 as a threshold) are highlighted in bold; CI = credible interval in this and subsequent models.

| **Fixed Effects** | Z-score | Lower 95% CI | Upper 95% CI | pMCMC |
| --- | --- | --- | --- | --- |
| Temperature (°) | 0.3029 | -7.5124 | 1.3552 | 0.108 |
| PC1 | 0.0670 | -0.1732 | 0.3103 | 0.618 |
| Mid-Range Latitude (°) | -0.4190 | -0.6806 | -0.1920 | **0.002** |

**Table S3.** Predictors of darker torso pelage within Order Diprotodontia.

| **Fixed Effects** | Z-score | Lower 95% CI | Upper 95% CI | pMCMC |
| --- | --- | --- | --- | --- |
| Temperature (°) | -0.3551 | -2.1779 | 1.3348 | 0.642 |
| PC1 | -0.2489 | -2.2519 | 1.6907 | 0.784 |
| Mid-Range Latitude (°) | -2.3402 | -4.8334 | -0.3989 | **0.012** |

**Table S4.** Predictors of darker torso pelage within Order Rodentia.

| **Fixed Effects** | Z-score | Lower 95% CI | Upper 95% CI | pMCMC |  |
| --- | --- | --- | --- | --- | --- |
| Temperature (°) | | 1.0928 | 0.1326 | 2.2123 | **0.024** |
| PC1 | 0.1977 | -0.2644 | 0.6965 | 0.430 |  |
| Mid-Range Latitude (°) | -0.5511 | -0.9921 | -0.1102 | **0.006** |  |

**Table S5.** Predictors of darker leg pelage across the mammal Class.

| **Fixed Effects** | Z-score | Lower 95% CI | Upper 95% CI | pMCMC |  |
| --- | --- | --- | --- | --- | --- |
| Temperature (°) | | 0.0518 | -0.2471 | 0.3597 | 0.736 |
| PC1 | 0.0019 | -0.2204 | 0.2038 | 0.994 |  |
| Mid-Range Latitude (°) | -0.4939 | -0.7328 | -0.2489 | **<0.001** |  |

**Table S6.** Predictors of darker leg pelage within Order Diprotodontia.

| **Fixed Effects** | Z-score | Lower 95% CI | Upper 95% CI | pMCMC |  |
| --- | --- | --- | --- | --- | --- |
| Temperature (°) | | -0.7945 | -2.6251 | 1.0611 | 0.366 |
| PC1 | -1.1651 | 3.1885 | 0.5754 | 0.168 |  |
| Mid-Range Latitude (°) | -2.5937 | -5.0156 | -0.5130 | **0.004** |  |

**Table S7.** Predictors of darker leg pelage within Order Rodentia.

| **Fixed Effects** | Z-score | Lower 95% CI | Upper 95% CI | pMCMC |  |
| --- | --- | --- | --- | --- | --- |
| Temperature (°) | | 0.1046 | -0.5558 | 0.8027 | 0.794 |
| PC1 | 0.1210 | -0.4105 | 0.6897 | 0.680 |  |
| Mid-Range Latitude (°) | -0.7344 | -1.2851 | -0.2017 | **<0.001** |  |

**Table S8.** Predictors of darker head pelage across the mammal Class.

| **Fixed Effects** | Z-score | Lower 95% CI | Upper 95% CI | pMCMC |  |
| --- | --- | --- | --- | --- | --- |
| Temperature (°) | | 0.0870 | -0.1917 | 0.4250 | 0.590 |
| PC1 | 0.3264 | 0.0818 | 0.5816 | **0.016** |  |
| Mid-Range Latitude (°) | -0.3677 | -0.6263 | -0.1137 | **0.008** |  |

**Table S9.** Predictors of darker head pelage within Order Rodentia.

| **Fixed Effects** | Z-score | Lower 95% CI | Upper 95% CI | pMCMC |  |
| --- | --- | --- | --- | --- | --- |
| Temperature (°) | | -0.0638 | -0.4689 | 0.3928 | 0.702 |
| PC1 | 0.4961 | 0.0440 | 1.0623 | **0.044** |  |
| Mid-Range Latitude (°) | -0.6074 | -0.9621 | -0.2694 | **0.002** |  |

**Table S10.** Predictors of darker tail pelage within Order Cetartiodactyla.

| **Fixed Effects** | Z-score | Lower 95% CI | Upper 95% CI | pMCMC |  |
| --- | --- | --- | --- | --- | --- |
| Temperature (°) | | 0.4383 | -0.4040 | 1.4619 | 0.330 |
| PC1 | 0.1138 | -0.5681 | 0.9275 | 0.726 |  |
| Mid-Range Latitude (°) | -0.8731 | -1.9758 | -0.0089 | **0.040** |  |

**Table S11.** Predictors of darker tail pelage across the mammal Class.

| **Fixed Effects** | Z-score | Lower 95% CI | Upper 95% CI | pMCMC |
| --- | --- | --- | --- | --- |
| Temperature (°) | 0.2762 | 0.0076 | 0.5213 | **0.028** |
| PC1 | 0.1443 | -0.0455 | 0.3363 | 0.142 |
| Mid-Range Latitude (°) | -0.1159 | -0.3469 | 0.0832 | 0.300 |

Significant red pelage results

**Table S12.** Predictors of redder tail pelage within Order Primates.

| **Fixed Effects** | Z-score | Lower 95% CI | Upper 95% CI | pMCMC |  |
| --- | --- | --- | --- | --- | --- |
| Temperature (°) | | 0.2798 | -0.4111 | 1.0009 | 0.468 |
| Mean Precipitation (mm/m) | 0.9421 | 0.0749 | 1.9423 | **0.022** |  |
| Mean Evapotranspiration (mm/m) | -0.4267 | -1.4247 | 0.4574 | 0.344 |  |
| Mid-Range Latitude (°) | 0.0419 | -0.5787 | 0.6586 | 0.950 |  |

Significant colourful pelage results

**Table S13.** Predictors of colourful torso pelage within Order Primates.

| **Fixed Effects** | Z-score | Lower 95% CI | Upper 95% CI | pMCMC |  |
| --- | --- | --- | --- | --- | --- |
| Temperature (°) | | -0.1496 | -0.6682 | 0.3772 | 0.554 |
| Mean Precipitation (mm/m) | 0.2422 | -0.4784 | 0.9512 | 0.504 |  |
| Mean Evapotranspiration (mm/m) | -0.6765 | -1.4926 | 0.1951 | 0.128 |  |
| Mid-Range Latitude (°) | -0.7765 | -1.3518 | -0.1923 | **0.010** |  |

**Table S14.** Predictors of colourful torso pelage within Order Rodentia.

| **Fixed Effects** | Z-score | Lower 95% CI | Upper 95% CI | pMCMC |  |
| --- | --- | --- | --- | --- | --- |
| Temperature (°) | | 0.5052 | 0.0574 | 0.97442 | **0.032** |
| PC1 | 0.2991 | -0.0980 | 0.7664 | 0.194 |  |
| Mid-Range Latitude (°) | 0.8295 | 0.3167 | 1.3870 | **<0.001** |  |

**Table S15.** Predictors of colourful torso pelage within Order Dasyuromorphia.

| **Fixed Effects** | Z-score | Lower 95% CI | Upper 95% CI | pMCMC |  |
| --- | --- | --- | --- | --- | --- |
| Temperature (°) | | 1.1062 | -0.8992 | 3.1755 | 0.292 |
| PC1 | 2.2237 | 0.3395 | 4.0565 | **0.020** |  |
| Mid-Range Latitude (°) | -0.9794 | -3.9541 | 1.9216 | 0.550 |  |

**Table S16.** Predictors of colourful head pelage across the mammal Class.

| **Fixed Effects** | Z-score | Lower 95% CI | Upper 95% CI | pMCMC |  |
| --- | --- | --- | --- | --- | --- |
| Temperature (°) | | 0.0850 | -0.1590 | 0.3333 | 0.528 |
| PC1 | 0.0692 | -0.1241 | 0.2942 | 0.524 |  |
| Mid-Range Latitude (°) | 0.2867 | 0.0340 | 0.5158 | **0.018** |  |

**Table S17.** Predictors of colourful head pelage within Order Rodentia.

| **Fixed Effects** | Z-score | Lower 95% CI | Upper 95% CI | pMCMC |  |
| --- | --- | --- | --- | --- | --- |
| Temperature (°) | | 0.0356 | -0.4746 | 0.4862 | 0.850 |
| PC1 | 0.0808 | -0.3361 | 0.5244 | 0.686 |  |
| Mid-Range Latitude (°) | 0.4779 | 0.0491 | 0.8635 | **0.018** |  |

**Table S18.** Predictors of colourful tail pelage within Order Primates.

| **Fixed Effects** | Z-score | Lower 95% CI | Upper 95% CI | pMCMC |
| --- | --- | --- | --- | --- |
| Temperature (°) | 0.6475 | 0.0439 | 1.3452 | **0.034** |
| Mean Precipitation (mm/m) | -0.3264 | -0.9750 | 0.2073 | 0.284 |
| Mean Evapotranspiration (mm/m) | 0.2585 | -0.3672 | 0.9539 | 0.482 |
| Mid-Range Latitude (°) | -0.0174 | -0.4986 | 0.4114 | 0.956 |

Significant contrasting pelage results

**Table S19.** Predictors of contrasting torso pelage across the mammal Class.

| **Fixed Effects** | Z-score | Lower 95% CI | Upper 95% CI | pMCMC |  |
| --- | --- | --- | --- | --- | --- |
| Temperature (°) | | -0.1580 | -0.3871 | 0.0714 | 0.184 |
| PC1 | 0.1158 | -0.0435 | 0.2560 | 0.152 |  |
| Mid-Range Latitude (°) | 0.2640 | 0.0757 | 0.4622 | **0.006** |  |

**Table S20.** Predictors of contrasting torso pelage within Order Dasyuromorphia.

| **Fixed Effects** | Z-score | Lower 95% CI | Upper 95% CI | pMCMC |  |
| --- | --- | --- | --- | --- | --- |
| Temperature (°) | | -0.9709 | -3.3682 | 1.5332 | 0.456 |
| PC1 | 0.3469 | -1.6080 | 2.4467 | 0.664 |  |
| Mid-Range Latitude (°) | 3.0051 | 0.8578 | 5.9065 | **0.006** |  |

**Table S21.** Predictors of contrasting leg pelage across the mammal Class.

| **Fixed Effects** | Z-score | Lower 95% CI | Upper 95% CI | pMCMC |  |
| --- | --- | --- | --- | --- | --- |
| Temperature (°) | | 0.0176 | -0.2491 | 0.2967 | 0.888 |
| PC1 | 0.0987 | -0.1317 | 0.3149 | 0.390 |  |
| Mid-Range Latitude (°) | 0.3604 | 0.0904 | 0.5956 | **0.002** |  |

**Table S22.** Predictors of contrasting leg pelage within Order Rodentia.

| **Fixed Effects** | Z-score | Lower 95% CI | Upper 95% CI | pMCMC |  |
| --- | --- | --- | --- | --- | --- |
| Temperature (°) | | 0.1531 | -0.2752 | 0.5510 | 0.0494 |
| PC1 | 0.2081 | -0.1136 | 0.5840 | 0.236 |  |
| Mid-Range Latitude (°) | 0.4767 | 0.1109 | 0.8744 | **0.020** |  |

**Table S23.** Predictors of contrasting head pelage within Order Rodentia.

| **Fixed Effects** | Z-score | Lower 95% CI | Upper 95% CI | pMCMC |  |
| --- | --- | --- | --- | --- | --- |
| Temperature (°) | | -0.0637 | -0.3709 | 0.1886 | 0.658 |
| PC1 | 0.2423 | 0.0269 | 0.4498 | **0.026** |  |
| Mid-Range Latitude (°) | 0.1565 | -0.1100 | 0.4033 | 0.254 |  |

Significant patterned pelage results

**Table S24.** Predictors of patterned torso coloration within Order Primates.

| **Fixed Effects** | Z-score | Lower 95% CI | Upper 95% CI | pMCMC |  |
| --- | --- | --- | --- | --- | --- |
| Temperature (°) | | -0.1559 | -0.6401 | 0.3130 | 0.512 |
| Mean Precipitation (mm/m) | 0.2366 | -0.4548 | 1.0073 | 0.534 |  |
| Mean Evapotranspiration (mm/m) | -0.4747 | -1.3119 | 0.3408 | 0.266 |  |
| Mid-Range Latitude (°) | -0.6973 | -1.3802 | -0.1197 | **0.018** |  |

**Table S25.** Predictors of patterned torso coloration within Order Rodentia.

| **Fixed Effects** | Z-score | Lower 95% CI | Upper 95% CI | pMCMC |  |
| --- | --- | --- | --- | --- | --- |
| Temperature (°) | | 0.3950 | 0.0007 | 0.7802 | 0.050 |
| PC1 | 0.2628 | -0.0778 | 0.6820 | 0.182 |  |
| Mid-Range Latitude (°) | 0.6591 | 0.3090 | 1.0989 | **<0.001** |  |

**Table S26.** Predictors of patterned torso coloration across the mammal Class.

| **Fixed Effects** | Z-score | Lower 95% CI | Upper 95% CI | pMCMC |  |
| --- | --- | --- | --- | --- | --- |
| Temperature (°) | | 0.0049 | -0.2232 | 0.2109 | 0.944 |
| PC1 | 0.1721 | -0.0008 | 0.3415 | **0.048** |  |
| Mid-Range Latitude (°) | 0.1681 | -0.0083 | 0.3860 | 0.086 |  |

**Table S27.** Predictors of patterned head coloration across the mammal Class.

| **Fixed Effects** | Z-score | Lower 95% CI | Upper 95% CI | pMCMC |  |
| --- | --- | --- | --- | --- | --- |
| Temperature (°) | | 0.0770 | -0.1727 | 0.3341 | 0.540 |
| PC1 | 0.0693 | -0.1336 | 0.2766 | 0.480 |  |
| Mid-Range Latitude (°) | 0.2871 | 0.0384 | 0.5363 | **0.032** |  |

**Table S28.** Predictors of patterned head coloration within Order Rodentia.

| **Fixed Effects** | Z-score | Lower 95% CI | Upper 95% CI | pMCMC |  |
| --- | --- | --- | --- | --- | --- |
| Temperature (°) | | 0.0347 | -0.5040 | 0.5274 | 0.848 |
| PC1 | 0.0879 | -0.3753 | 0.4910 | 0.664 |  |
| Mid-Range Latitude (°) | 0.4686 | 0.0165 | 0.8840 | **0.032** |  |

**Table S29.** Predictors of patterned tail coloration within Order Primates.

| **Fixed Effects** | Z-score | Lower 95% CI | Upper 95% CI | pMCMC |
| --- | --- | --- | --- | --- |
| Temperature (°) | 0.6583 | 0.0634 | 1.4051 | **0.018** |
| Mean Precipitation (mm/m) | -0.2992 | -0.8345 | 0.2907 | 0.312 |
| Mean Evapotranspiration (mm/m) | 0.2386 | -0.4520 | 0.9294 | 0.482 |
| Mid-Range Latitude (°) | -0.0174 | -0.4614 | 0.4049 | 0.946 |

Non-significant dark pelage results

**Table S30.** Predictors of darker torso pelage within Order Carnivora.

| **Fixed Effects** | Z-score | Lower 95% CI | Upper 95% CI | pMCMC |  |
| --- | --- | --- | --- | --- | --- |
| Temperature (°) | | 0.4218 | -1.4106 | 2.0097 | 0.630 |
| PC1 | -0.1201 | -1.4406 | 1.2962 | 0.858 |  |
| Mid-Range Latitude (°) | -1.0710 | -2.6429 | 0.4503 | 0.162 |  |

**Table S31.** Predictors of darker torso pelage within Order Cetartiodactyla.

| **Fixed Effects** | Z-score | Lower 95% CI | Upper 95% CI | pMCMC |
| --- | --- | --- | --- | --- |
| Temperature (°) | 1.0034 | -0.4499 | 2.5898 | 0.162 |
| PC1 | -0.2952 | -1.4626 | 0.5937 | 0.494 |
| Mid-Range Latitude (°) | 0.4646 | -0.6817 | 2.0534 | 0.526 |

**Table S32.** Predictors of darker torso pelage within Order Dasyuromorphia.

| **Fixed Effects** | Z-score | Lower 95% CI | Upper 95% CI | pMCMC |
| --- | --- | --- | --- | --- |
| Temperature (°) | -0.9885 | -3.6102 | 1.5237 | 0.374 |
| PC1 | 0.4910 | -2.7624 | 3.4912 | 0.736 |
| Mid-Range Latitude (°) | -0.7920 | -3.4207 | 1.5491 | 0.422 |

**Table S33.** Predictors of darker torso pelage within Order Eulipotyphla.

| **Fixed Effects** | Z-score | Lower 95% CI | Upper 95% CI | pMCMC |
| --- | --- | --- | --- | --- |
| Temperature (°) | 0.2780 | -0.5672 | 1.3171 | 0.634 |
| Mean Precipitation (mm/m) | -0.4151 | -2.0818 | 1.1200 | 0.592 |
| Mean Evapotranspiration (mm/m) | 0.4322 | -1.3590 | 2.2626 | 0.656 |
| Mid-Range Latitude (°) | 0.3823 | -0.8180 | 1.7048 | 0.558 |

**Table S34.** Predictors of darker torso pelage within Order Primates.

| **Fixed Effects** | Z-score | Lower 95% CI | Upper 95% CI | pMCMC |
| --- | --- | --- | --- | --- |
| Temperature (°) | -0.1956 | -0.7807 | 0.4080 | 0.466 |
| Mean Precipitation (mm/m) | 0.0188 | -0.8582 | 1.0205 | 1.000 |
| Mean Evapotranspiration (mm/m) | 0.3497 | -0.7288 | 1.4391 | 0.514 |
| Mid-Range Latitude (°) | -0.5693 | -1.4840 | 0.2478 | 0.150 |

**Table S35.** Predictors of darker leg pelage within Order Carnivora.

| **Fixed Effects** | Z-score | Lower 95% CI | Upper 95% CI | pMCMC |
| --- | --- | --- | --- | --- |
| Temperature (°) | -0.0938 | -1.1258 | 0.8864 | 0.838 |
| PC1 | -0.5309 | -1.3658 | 0.1506 | 0.124 |
| Mid-Range Latitude (°) | -0.4842 | -1.4786 | 0.4562 | 0.260 |

**Table S36.** Predictors of darker leg pelage within Order Cetartiodactyla.

| **Fixed Effects** | Z-score | Lower 95% CI | Upper 95% CI | pMCMC |
| --- | --- | --- | --- | --- |
| Temperature (°) | 0.2112 | -0.7356 | 1.1455 | 0.696 |
| PC1 | 0.1076 | -0.6261 | 1.0051 | 0.894 |
| Mid-Range Latitude (°) | -0.4493 | -1.4015 | 0.3114 | 0.232 |

**Table S37.** Predictors of darker leg pelage within Order Dasyuromorphia.

| **Fixed Effects** | Z-score | Lower 95% CI | Upper 95% CI | pMCMC |
| --- | --- | --- | --- | --- |
| Temperature (°) | -1.8239 | -4.2588 | 0.6678 | 0.130 |
| PC1 | 0.1900 | -2.7009 | 3.2697 | 0.950 |
| Mid-Range Latitude (°) | -0.8174 | -3.1104 | 1.2092 | 0.436 |

**Table S38.** Predictors of darker leg pelage within Order Eulipotyphla.

| **Fixed Effects** | Z-score | Lower 95% CI | Upper 95% CI | pMCMC |
| --- | --- | --- | --- | --- |
| Temperature (°) | 0.3485 | -1.0740 | 1.8443 | 0.698 |
| Mean Precipitation (mm/m) | -1.2847 | -3.2694 | 0.6972 | 0.200 |
| Mean Evapotranspiration (mm/m) | 1.8988 | -0.1106 | 4.0770 | 0.078 |
| Mid-Range Latitude (°) | -0.0562 | -1.5586 | 1.6042 | 0.970 |

**Table S39.** Predictors of darker leg pelage within Order Primates.

| **Fixed Effects** | Z-score | Lower 95% CI | Upper 95% CI | pMCMC |
| --- | --- | --- | --- | --- |
| Temperature (°) | -0.0881 | -0.6103 | 0.4519 | 0.740 |
| Mean Precipitation (mm/m) | 0.1793 | -0.7391 | 1.0135 | 0.662 |
| Mean Evapotranspiration (mm/m) | 0.4098 | -0.5995 | 1.5938 | 0.464 |
| Mid-Range Latitude (°) | -0.2149 | -0.9681 | 0.4379 | 0.540 |

**Table S40.** Predictors of darker head pelage within Order Carnivora.

| **Fixed Effects** | Z-score | Lower 95% CI | Upper 95% CI | pMCMC |
| --- | --- | --- | --- | --- |
| Temperature (°) | 0.7765 | -0.9887 | 2.6989 | 0.362 |
| PC1 | -0.2179 | -1.7523 | 1.4106 | 0.746 |
| Mid-Range Latitude (°) | -0.3409 | -2.1407 | 1.7658 | 0.722 |

**Table S41.** Predictors of darker head pelage within Order Cetartiodactyla.

| **Fixed Effects** | Z-score | Lower 95% CI | Upper 95% CI | pMCMC |
| --- | --- | --- | --- | --- |
| Temperature (°) | 1.2008 | -0.6386 | 3.4780 | 0.204 |
| PC1 | 0.7317 | -0.9201 | 2.6043 | 0.388 |
| Mid-Range Latitude (°) | -0.2772 | -1.9242 | 1.2725 | 0.726 |

**Table S42.** Predictors of darker head pelage within Order Dasyuromorphia.

| **Fixed Effects** | Z-score | Lower 95% CI | Upper 95% CI | pMCMC |
| --- | --- | --- | --- | --- |
| Temperature (°) | -1.9729 | -4.7077 | 1.1160 | 0.190 |
| PC1 | 0.3748 | -3.0942 | 3.6453 | 0.826 |
| Mid-Range Latitude (°) | -0.4127 | -3.3405 | 2.3601 | 0.746 |

**Table S43.** Predictors of darker head pelage within Order Diprotodontia.

| **Fixed Effects** | Z-score | Lower 95% CI | Upper 95% CI | pMCMC |
| --- | --- | --- | --- | --- |
| Temperature (°) | -0.3454 | -1.7361 | 1.0411 | 0.604 |
| PC1 | -0.2727 | -1.7905 | 1.4324 | 0.650 |
| Mid-Range Latitude (°) | -1.2460 | -2.7819 | 0.5250 | 0.090 |

**Table S44.** Predictors of darker head pelage within Order Eulipotyphla.

| **Fixed Effects** | Z-score | Lower 95% CI | Upper 95% CI | pMCMC |
| --- | --- | --- | --- | --- |
| Temperature (°) | 0.2219 | -1.0898 | 2.2308 | 0.878 |
| Mean Precipitation (mm/m) | -0.4928 | -2.7536 | 1.5442 | 0.640 |
| Mean Evapotranspiration (mm/m) | 0.4149 | -1.6505 | 3.0965 | 0.758 |
| Mid-Range Latitude (°) | -0.2573 | -2.0421 | 1.4765 | 0.762 |

**Table S45.** Predictors of darker head pelage within Order Primates.

| **Fixed Effects** | Z-score | Lower 95% CI | Upper 95% CI | pMCMC |
| --- | --- | --- | --- | --- |
| Temperature (°) | 0.1106 | -0.5158 | 0.7405 | 0.802 |
| Mean Precipitation (mm/m) | 0.7731 | -0.1351 | 1.7220 | 0.088 |
| Mean Evapotranspiration (mm/m) | 0.0673 | -1.0056 | 1.2254 | 0.898 |
| Mid-Range Latitude (°) | -0.0700 | -0.7521 | 0.6038 | 0.864 |

**Table S46.** Predictors of darker tail pelage within Order Carnivora.

| **Fixed Effects** | Z-score | Lower 95% CI | Upper 95% CI | pMCMC |
| --- | --- | --- | --- | --- |
| Temperature (°) | 0.8021 | -0.4856 | 2.1798 | 0.224 |
| PC1 | 0.1563 | -0.6651 | 1.0661 | 0.736 |
| Mid-Range Latitude (°) | -0.1705 | -1.3493 | 0.8432 | 0.716 |

**Table S47.** Predictors of darker tail pelage within Order Dasyuromorphia.

| **Fixed Effects** | Z-score | Lower 95% CI | Upper 95% CI | pMCMC |
| --- | --- | --- | --- | --- |
| Temperature (°) | -1.4842 | -4.3609 | 0.9931 | 0.250 |
| PC1 | 0.6956 | -2.3491 | 3.8916 | 0.676 |
| Mid-Range Latitude (°) | -0.5701 | -3.1654 | 2.0324 | 0.622 |

**Table S48.** Predictors of darker tail pelage within Order Diprotodontia.

| **Fixed Effects** | Z-score | Lower 95% CI | Upper 95% CI | pMCMC |
| --- | --- | --- | --- | --- |
| Temperature (°) | -1.0796 | -3.2942 | 0.8250 | 0.260 |
| PC1 | 0.8389 | -1.6718 | 3.2830 | 0.510 |
| Mid-Range Latitude (°) | -1.7587 | -4.1293 | 0.5705 | 0.116 |

**Table S49.** Predictors of darker tail pelage within Order Eulipotyphla.

| **Fixed Effects** | Z-score | Lower 95% CI | Upper 95% CI | pMCMC |
| --- | --- | --- | --- | --- |
| Temperature (°) | 0.8793 | -0.9609 | 3.0026 | 0.370 |
| Mean Precipitation (mm/m) | -0.1187 | -2.3264 | 2.1923 | 0.938 |
| Mean Evapotranspiration (mm/m) | 1.3756 | -1.0443 | 3.8722 | 0.250 |
| Mid-Range Latitude (°) | 0.1244 | -2.2320 | 1.9384 | 0.828 |

**Table S50.** Predictors of darker tail pelage within Order Primates.

| **Fixed Effects** | Z-score | Lower 95% CI | Upper 95% CI | pMCMC |
| --- | --- | --- | --- | --- |
| Temperature (°) | -0.1450 | -0.6361 | 0.3441 | 0.522 |
| Mean Precipitation (mm/m) | -0.2590 | -1.0797 | 0.4540 | 0.522 |
| Mean Evapotranspiration (mm/m) | 0.4120 | -0.5011 | 1.2633 | 0.348 |
| Mid-Range Latitude (°) | 0.1395 | -0.4166 | 0.7579 | 0.630 |

**Table S51.** Predictors of darker tail pelage within Order Rodentia.

| **Fixed Effects** | Z-score | Lower 95% CI | Upper 95% CI | pMCMC |
| --- | --- | --- | --- | --- |
| Temperature (°) | 0.3592 | -0.0863 | 0.8022 | 0.092 |
| PC1 | -0.0388 | -0.3173 | 0.2846 | 0.798 |
| Mid-Range Latitude (°) | 0.0609 | -0.2359 | 0.4408 | 0.744 |

Non-significant red pelage results

**Table S52.** Predictors of redder torso pelage across the mammal Class.

| **Fixed Effects** | Z-score | Lower 95% CI | Upper 95% CI | pMCMC |
| --- | --- | --- | --- | --- |
| Temperature (°) | 0.0027 | -0.1562 | 0.1659 | 0.964 |
| PC1 | 0.0342 | -0.1021 | 0.1684 | 0.634 |
| Mid-Range Latitude (°) | -0.1163 | -0.2662 | 0.0521 | 0.172 |

**Table S53.** Predictors of redder torso pelage within Order Carnivora.

| **Fixed Effects** | Z-score | Lower 95% CI | Upper 95% CI | pMCMC |
| --- | --- | --- | --- | --- |
| Temperature (°) | -0.1039 | -0.5877 | 0.4071 | 0.660 |
| PC1 | -0.2186 | -0.6508 | 0.2335 | 0.348 |
| Mid-Range Latitude (°) | 0.1702 | -0.3597 | 0.7031 | 0.530 |

**Table S54.** Predictors of redder torso pelage within Order Cetartiodactyla.

| **Fixed Effects** | Z-score | Lower 95% CI | Upper 95% CI | pMCMC |
| --- | --- | --- | --- | --- |
| Temperature (°) | 0.7919 | -1.0540 | 2.6069 | 0.356 |
| PC1 | 0.3595 | -1.1920 | 1.8280 | 0.632 |
| Mid-Range Latitude (°) | -0.7393 | -2.6018 | 1.0659 | 0.366 |

**Table S55.** Predictors of redder torso pelage within Order Dasyuromorphia.

| **Fixed Effects** | Z-score | Lower 95% CI | Upper 95% CI | pMCMC |
| --- | --- | --- | --- | --- |
| Temperature (°) | 0.3409 | -2.5465 | 3.2397 | 0.790 |
| PC1 | 1.1723 | -1.3614 | 4.2068 | 0.322 |
| Mid-Range Latitude (°) | -0.9652 | -4.3478 | 1.8866 | 0.496 |

**Table S56.** Predictors of redder torso pelage within Order Diprotodontia.

| **Fixed Effects** | Z-score | Lower 95% CI | Upper 95% CI | pMCMC |
| --- | --- | --- | --- | --- |
| Temperature (°) | 0.9088 | -0.3982 | 2.1618 | 0.134 |
| PC1 | 0.2137 | -0.8903 | 1.2406 | 0.694 |
| Mid-Range Latitude (°) | 0.7975 | -0.7569 | 2.3340 | 0.256 |

**Table S57.** Predictors of redder torso pelage within Order Eulipotyphla.

| **Fixed Effects** | Z-score | Lower 95% CI | Upper 95% CI | pMCMC |
| --- | --- | --- | --- | --- |
| Temperature (°) | 0.2196 | -0.5742 | 1.1223 | 0.640 |
| Mean Precipitation (mm/m) | -0.2502 | -1.8891 | 1.2394 | 0.716 |
| Mean Evapotranspiration (mm/m) | 0.0279 | -1.6781 | 2.0643 | 0.926 |
| Mid-Range Latitude (°) | 0.5071 | -0.7674 | 1.7417 | 0.454 |

**Table S58.** Predictors of redder torso pelage within Order Primates.

| **Fixed Effects** | Z-score | Lower 95% CI | Upper 95% CI | pMCMC |
| --- | --- | --- | --- | --- |
| Temperature (°) | -0.1597 | -0.5285 | 0.2688 | 0.442 |
| Mean Precipitation (mm/m) | 0.1398 | -0.5135 | 0.7521 | 0.656 |
| Mean Evapotranspiration (mm/m) | 0.6581 | -0.1129 | 1.3927 | 0.084 |
| Mid-Range Latitude (°) | 0.0125 | -0.4940 | 0.4517 | 0.990 |

**Table S59.** Predictors of redder torso pelage within Order Rodentia.

| **Fixed Effects** | Z-score | Lower 95% CI | Upper 95% CI | pMCMC |
| --- | --- | --- | --- | --- |
| Temperature (°) | -0.0173 | -0.2651 | 0.2532 | 0.918 |
| PC1 | -0.1631 | -0.4006 | 0.0504 | 0.150 |
| Mid-Range Latitude (°) | -0.1608 | -0.4336 | 0.1016 | 0.210 |

**Table S60.** Predictors of redder leg pelage across the mammal Class.

| **Fixed Effects** | Z-score | Lower 95% CI | Upper 95% CI | pMCMC |
| --- | --- | --- | --- | --- |
| Temperature (°) | 0.1056 | -0.0771 | 0.3013 | 0.296 |
| PC1 | 0.1439 | -0.0166 | 0.3071 | 0.080 |
| Mid-Range Latitude (°) | -0.1454 | -0.3188 | 0.0391 | 0.116 |

**Table S61.** Predictors of redder leg pelage within Order Carnivora.

| **Fixed Effects** | Z-score | Lower 95% CI | Upper 95% CI | pMCMC |
| --- | --- | --- | --- | --- |
| Temperature (°) | 0.0220 | -0.5965 | 0.7582 | 0.978 |
| PC1 | 0.1687 | -0.4268 | 0.8448 | 0.582 |
| Mid-Range Latitude (°) | 0.0971 | -0.5540 | 0.8299 | 0.800 |

**Table S62.** Predictors of redder leg pelage within Order Cetartiodactyla.

| **Fixed Effects** | Z-score | Lower 95% CI | Upper 95% CI | pMCMC |
| --- | --- | --- | --- | --- |
| Temperature (°) | 0.8170 | -0.3135 | 2.2815 | 0.138 |
| PC1 | -0.0759 | -0.8351 | 0.7591 | 0.814 |
| Mid-Range Latitude (°) | -0.2133 | -1.1843 | 0.7396 | 0.598 |

**Table S63.** Predictors of redder leg pelage within Order Dasyuromorphia.

| **Fixed Effects** | Z-score | Lower 95% CI | Upper 95% CI | pMCMC |
| --- | --- | --- | --- | --- |
| Temperature (°) | 0.4128 | -2.2727 | 3.1737 | 0.750 |
| PC1 | 0.8315 | -1.8487 | 3.4837 | 0.430 |
| Mid-Range Latitude (°) | -1.2133 | -3.7727 | 1.4574 | 0.322 |

**Table S64.** Predictors of redder leg pelage within Order Diprotodontia.

| **Fixed Effects** | Z-score | Lower 95% CI | Upper 95% CI | pMCMC |
| --- | --- | --- | --- | --- |
| Temperature (°) | 1.0437 | -0.7104 | 2.8718 | 0.192 |
| PC1 | -0.1179 | -1.6939 | 1.3404 | 0.866 |
| Mid-Range Latitude (°) | 0.7994 | -1.2901 | 3.0546 | 0.388 |

**Table S65.** Predictors of redder leg pelage within Order Eulipotyphla.

| **Fixed Effects** | Z-score | Lower 95% CI | Upper 95% CI | pMCMC |
| --- | --- | --- | --- | --- |
| Temperature (°) | 0.0704 | -0.8835 | 0.8881 | 0.872 |
| Mean Precipitation (mm/m) | -0.7901 | -2.4275 | 0.9016 | 0.298 |
| Mean Evapotranspiration (mm/m) | 0.3566 | -1.6837 | 2.2189 | 0.666 |
| Mid-Range Latitude (°) | 0.2123 | -1.3088 | 1.7626 | 0.740 |

**Table S66.** Predictors of redder leg pelage within Order Primates.

| **Fixed Effects** | Z-score | Lower 95% CI | Upper 95% CI | pMCMC |
| --- | --- | --- | --- | --- |
| Temperature (°) | -0.1386 | -0.5149 | 0.2474 | 0.460 |
| Mean Precipitation (mm/m) | 0.1233 | -0.5589 | 0.6888 | 0.714 |
| Mean Evapotranspiration (mm/m) | 0.3767 | -0.2718 | 1.1181 | 0.262 |
| Mid-Range Latitude (°) | -0.0536 | -0.5274 | 0.3825 | 0.790 |

**Table S67.** Predictors of redder leg pelage within Order Rodentia.

| **Fixed Effects** | Z-score | Lower 95% CI | Upper 95% CI | pMCMC |
| --- | --- | --- | --- | --- |
| Temperature (°) | 0.1222 | -0.1651 | 0.4437 | 0.446 |
| PC1 | 0.0768 | -0.1574 | 0.3451 | 0.550 |
| Mid-Range Latitude (°) | -0.1951 | -0.4786 | 0.0661 | 0.166 |

**Table S68.** Predictors of redder head pelage across the mammal Class.

| **Fixed Effects** | Z-score | Lower 95% CI | Upper 95% CI | pMCMC |
| --- | --- | --- | --- | --- |
| Temperature (°) | 0.1086 | -0.0610 | 0.2879 | 0.236 |
| PC1 | -0.0101 | -0.1462 | 0.1314 | 0.894 |
| Mid-Range Latitude (°) | -0.0748 | -0.2535 | 0.1121 | 0.420 |

**Table S69.** Predictors of redder head pelage within Order Carnivora.

| **Fixed Effects** | Z-score | Lower 95% CI | Upper 95% CI | pMCMC |
| --- | --- | --- | --- | --- |
| Temperature (°) | 0.0912 | -0.4918 | 0.7077 | 0.760 |
| PC1 | -0.0099 | -0.4915 | 0.5002 | 0.934 |
| Mid-Range Latitude (°) | -0.0880 | -0.6533 | 0.5488 | 0.784 |

**Table S70.** Predictors of redder head pelage within Order Cetartiodactyla.

| **Fixed Effects** | Z-score | Lower 95% CI | Upper 95% CI | pMCMC |
| --- | --- | --- | --- | --- |
| Temperature (°) | 0.3336 | -0.3192 | 1.0974 | 0.328 |
| PC1 | 0.0462 | -0.5025 | 0.7094 | 0.912 |
| Mid-Range Latitude (°) | -0.0532 | -0.8440 | 0.6592 | 0.872 |

**Table S71.** Predictors of redder head pelage within Order Dasyuromorphia.

| **Fixed Effects** | Z-score | Lower 95% CI | Upper 95% CI | pMCMC |
| --- | --- | --- | --- | --- |
| Temperature (°) | -0.1453 | -2.4141 | 2.7869 | 0.762 |
| PC1 | -0.6492 | -3.1094 | 1.9377 | 0.530 |
| Mid-Range Latitude (°) | -1.7916 | -5.0628 | 0.8395 | 0.150 |

**Table S72.** Predictors of redder head pelage within Order Diprotodontia.

| **Fixed Effects** | Z-score | Lower 95% CI | Upper 95% CI | pMCMC |
| --- | --- | --- | --- | --- |
| Temperature (°) | 0.1316 | -1.4273 | 1.7995 | 0.896 |
| PC1 | 0.1182 | -1.6684 | 1.5586 | 0.798 |
| Mid-Range Latitude (°) | -0.2228 | -2.0670 | 1.9456 | 0.806 |

**Table S73.** Predictors of redder head pelage within Order Eulipotyphla.

| **Fixed Effects** | Z-score | Lower 95% CI | Upper 95% CI | pMCMC |
| --- | --- | --- | --- | --- |
| Temperature (°) | 0.4481 | -0.5593 | 1.5217 | 0.358 |
| Mean Precipitation (mm/m) | -0.4250 | -2.1450 | 1.3713 | 0.594 |
| Mean Evapotranspiration (mm/m) | 0.0028 | -2.5351 | 1.8413 | 0.906 |
| Mid-Range Latitude (°) | 0.3445 | -1.1276 | 2.1781 | 0.656 |

**Table S74.** Predictors of redder head pelage within Order Primates.

| **Fixed Effects** | Z-score | Lower 95% CI | Upper 95% CI | pMCMC |
| --- | --- | --- | --- | --- |
| Temperature (°) | -0.1023 | -0.5597 | 0.4379 | 0.670 |
| Mean Precipitation (mm/m) | 0.2280 | -0.5637 | 0.9657 | 0.558 |
| Mean Evapotranspiration (mm/m) | 0.3498 | -0.5703 | 1.1757 | 0.420 |
| Mid-Range Latitude (°) | 0.0652 | -0.5782 | 0.6370 | 0.850 |

**Table S75.** Predictors of redder head pelage within Order Rodentia.

| **Fixed Effects** | Z-score | Lower 95% CI | Upper 95% CI | pMCMC |
| --- | --- | --- | --- | --- |
| Temperature (°) | 0.0667 | -0.1743 | 0.3464 | 0.628 |
| PC1 | -0.1129 | -0.3455 | 0.1100 | 0.336 |
| Mid-Range Latitude (°) | -0.1193 | -0.3979 | 0.1455 | 0.404 |

**Table S76.** Predictors of redder tail pelage across the mammal Class.

| **Fixed Effects** | Z-score | Lower 95% CI | Upper 95% CI | pMCMC |
| --- | --- | --- | --- | --- |
| Temperature (°) | 0.0594 | -0.1338 | 0.2522 | 0.558 |
| PC1 | 0.0868 | -0.0751 | 0.2705 | 0.344 |
| Mid-Range Latitude (°) | -0.0183 | -0.2042 | 0.1902 | 0.878 |

**Table S77.** Predictors of redder tail pelage within Order Carnivora.

| **Fixed Effects** | Z-score | Lower 95% CI | Upper 95% CI | pMCMC |
| --- | --- | --- | --- | --- |
| Temperature (°) | -0.0683 | -0.6937 | 0.5514 | 0.810 |
| PC1 | -0.1735 | -0.7278 | 0.3903 | 0.554 |
| Mid-Range Latitude (°) | 0.2941 | -0.3806 | 0.9387 | 0.376 |

**Table S78.** Predictors of redder tail pelage within Order Cetartiodactyla.

| **Fixed Effects** | Z-score | Lower 95% CI | Upper 95% CI | pMCMC |
| --- | --- | --- | --- | --- |
| Temperature (°) | 1.0440 | -0.0965 | 2.4881 | 0.056 |
| PC1 | 0.0321 | -0.8274 | 1.0673 | 0.960 |
| Mid-Range Latitude (°) | -0.1940 | -1.2066 | 0.9448 | 0.632 |

**Table S79.** Predictors of redder tail pelage within Order Dasyuromorphia.

| **Fixed Effects** | Z-score | Lower 95% CI | Upper 95% CI | pMCMC |
| --- | --- | --- | --- | --- |
| Temperature (°) | 0.6932 | -2.7652 | 3.5761 | 0.622 |
| PC1 | 0.3944 | -2.7406 | 4.0729 | 0.796 |
| Mid-Range Latitude (°) | 0.5430 | -2.7811 | 3.5346 | 0.700 |

**Table S80.** Predictors of redder tail pelage within Order Diprotodontia.

| **Fixed Effects** | Z-score | Lower 95% CI | Upper 95% CI | pMCMC |
| --- | --- | --- | --- | --- |
| Temperature (°) | 0.6213 | -1.4416 | 2.7618 | 0.506 |
| PC1 | -0.2791 | -2.1856 | 1.3813 | 0.778 |
| Mid-Range Latitude (°) | 0.5449 | -1.8213 | 3.3053 | 0.662 |

**Table S81.** Predictors of redder tail pelage within Order Eulipotyphla.

| **Fixed Effects** | Z-score | Lower 95% CI | Upper 95% CI | pMCMC |
| --- | --- | --- | --- | --- |
| Temperature (°) | 0.1137 | -0.7642 | 0.8803 | 0.762 |
| Mean Precipitation (mm/m) | -0.5659 | -2.0852 | 0.9330 | 0.448 |
| Mean Evapotranspiration (mm/m) | 0.1088 | -1.9371 | 1.8993 | 0.904 |
| Mid-Range Latitude (°) | 0.1726 | -1.1576 | 1.6553 | 0.812 |

**Table S82.** Predictors of redder tail pelage within Order Rodentia.

| **Fixed Effects** | Z-score | Lower 95% CI | Upper 95% CI | pMCMC |
| --- | --- | --- | --- | --- |
| Temperature (°) | -0.0566 | -0.3126 | 0.2406 | 0.704 |
| PC1 | 0.1015 | -0.1756 | 0.4154 | 0.518 |
| Mid-Range Latitude (°) | 0.0076 | -0.3261 | 0.3283 | 0.986 |

Non-significant colourful pelage results

**Table S83.** Predictors of colourful torso pelage across the mammal Class.

| **Fixed Effects** | Z-score | Lower 95% CI | Upper 95% CI | pMCMC |
| --- | --- | --- | --- | --- |
| Temperature (°) | 0.1107 | -0.1217 | 0.3494 | 0.350 |
| PC1 | 0.1789 | 0.0013 | 0.3895 | 0.068 |
| Mid-Range Latitude (°) | 0.1787 | -0.0189 | 0.3945 | 0.102 |

**Table S84.** Predictors of colourful torso pelage within Order Carnivora.

| **Fixed Effects** | Z-score | Lower 95% CI | Upper 95% CI | pMCMC |
| --- | --- | --- | --- | --- |
| Temperature (°) | 0.3716 | -0.4603 | 1.2153 | 0.334 |
| PC1 | -0.2174 | -1.0389 | 0.4722 | 0.606 |
| Mid-Range Latitude (°) | 0.1905 | -0.6216 | 0.9687 | 0.626 |

**Table S85.** Predictors of colourful torso pelage within Order Cetartiodactyla.

| **Fixed Effects** | Z-score | Lower 95% CI | Upper 95% CI | pMCMC |
| --- | --- | --- | --- | --- |
| Temperature (°) | 0.1737 | -0.7204 | 0.9278 | 0.670 |
| PC1 | -0.0138 | -0.7762 | 0.7132 | 0.914 |
| Mid-Range Latitude (°) | -0.1884 | -1.1050 | 0.6165 | 0.672 |

**Table S86.** Predictors of colourful torso pelage within Order Diprotodontia.

| **Fixed Effects** | Z-score | Lower 95% CI | Upper 95% CI | pMCMC |
| --- | --- | --- | --- | --- |
| Temperature (°) | -1.6673 | -3.9268 | 0.3396 | 0.112 |
| PC1 | -0.7143 | -2.5179 | 1.2729 | 0.460 |
| Mid-Range Latitude (°) | -0.4482 | -2.8901 | 1.7014 | 0.650 |

**Table S87.** Predictors of colourful torso pelage within Order Eulipotyphla.

| **Fixed Effects** | Z-score | Lower 95% CI | Upper 95% CI | pMCMC |
| --- | --- | --- | --- | --- |
| Temperature (°) | -0.2543 | -1.0486 | 0.3837 | 0.488 |
| Mean Precipitation (mm/m) | -1.0353 | -2.1681 | 0.0735 | 0.076 |
| Mean Evapotranspiration (mm/m) | -0.1155 | -1.3600 | 1.1823 | 0.866 |
| Mid-Range Latitude (°) | -0.1023 | -0.7406 | 0.5963 | 0.716 |

**Table S88.** Predictors of colourful leg pelage across the mammal Class.

| **Fixed Effects** | Z-score | Lower 95% CI | Upper 95% CI | pMCMC |
| --- | --- | --- | --- | --- |
| Temperature (°) | 0.1509 | -0.0588 | 0.3534 | 0.174 |
| PC1 | -0.0245 | -0.1702 | 0.1249 | 0.728 |
| Mid-Range Latitude (°) | -0.0406 | -0.2377 | 0.1561 | 0.654 |

**Table S89.** Predictors of colourful leg pelage within Order Carnivora.

| **Fixed Effects** | Z-score | Lower 95% CI | Upper 95% CI | pMCMC |
| --- | --- | --- | --- | --- |
| Temperature (°) | -0.0192 | -1.5392 | 1.3773 | 0.994 |
| PC1 | -0.8327 | -2.2311 | 0.4758 | 0.142 |
| Mid-Range Latitude (°) | 0.6602 | -0.5066 | 2.3167 | 0.336 |

**Table S90.** Predictors of colourful leg pelage within Order Cetartiodactyla.

| **Fixed Effects** | Z-score | Lower 95% CI | Upper 95% CI | pMCMC |
| --- | --- | --- | --- | --- |
| Temperature (°) | 0.4019 | -0.3280 | 1.2306 | 0.248 |
| PC1 | 0.1780 | -0.3443 | 0.8309 | 0.526 |
| Mid-Range Latitude (°) | -0.2548 | -0.9270 | 0.5658 | 0.518 |

**Table S91.** Predictors of colourful leg pelage within Order Dasyuromorphia.

| **Fixed Effects** | Z-score | Lower 95% CI | Upper 95% CI | pMCMC |
| --- | --- | --- | --- | --- |
| Temperature (°) | -0.1455 | -3.1752 | 2.5930 | 0.888 |
| PC1 | 1.4855 | -1.2257 | 4.8454 | 0.312 |
| Mid-Range Latitude (°) | -0.3322 | -3.2700 | 2.5328 | 0.792 |

**Table S92.** Predictors of colourful leg pelage within Order Diprotodontia.

| **Fixed Effects** | Z-score | Lower 95% CI | Upper 95% CI | pMCMC |
| --- | --- | --- | --- | --- |
| Temperature (°) | 0.9315 | -0.7645 | 3.1170 | 0.292 |
| PC1 | -1.5172 | -3.9176 | 0.3658 | 0.098 |
| Mid-Range Latitude (°) | 1.2696 | -1.0393 | 3.9310 | 0.254 |

**Table S93.** Predictors of colourful leg pelage within Order Eulipotyphla.

| **Fixed Effects** | Z-score | Lower 95% CI | Upper 95% CI | pMCMC |
| --- | --- | --- | --- | --- |
| Temperature (°) | 0.4096 | -1.0788 | 2.3212 | 0.670 |
| Mean Precipitation (mm/m) | 0.7547 | -1.3157 | 2.9624 | 0.498 |
| Mean Evapotranspiration (mm/m) | -0.5195 | -2.8317 | 2.0739 | 0.642 |
| Mid-Range Latitude (°) | -0.0961 | -1.6643 | 1.4079 | 0.906 |

**Table S94.** Predictors of colourful leg pelage within Order Primates.

| **Fixed Effects** | Z-score | Lower 95% CI | Upper 95% CI | pMCMC |
| --- | --- | --- | --- | --- |
| Temperature (°) | -0.1959 | -0.5947 | 0.2136 | 0.338 |
| Mean Precipitation (mm/m) | -0.1571 | -0.7168 | 0.4147 | 0.580 |
| Mean Evapotranspiration (mm/m) | 0.3973 | -0.2281 | 1.1590 | 0.226 |
| Mid-Range Latitude (°) | -0.0280 | -0.4812 | 0.3834 | 0.922 |

**Table S95.** Predictors of colourful leg pelage within Order Rodentia.

| **Fixed Effects** | Z-score | Lower 95% CI | Upper 95% CI | pMCMC |
| --- | --- | --- | --- | --- |
| Temperature (°) | 0.3010 | -0.0492 | 0.5986 | 0.084 |
| PC1 | -0.0854 | -0.3341 | 0.1608 | 0.462 |
| Mid-Range Latitude (°) | -0.0375 | -0.3789 | 0.2568 | 0.810 |

**Table S96.** Predictors of colourful head pelage within Order Carnivora.

| **Fixed Effects** | Z-score | Lower 95% CI | Upper 95% CI | pMCMC |  |
| --- | --- | --- | --- | --- | --- |
| Temperature (°) | | -1.5861 | -3.8831 | 0.5602 | 0.140 |
| PC1 | -1.1081 | -2.5174 | 0.1383 | 0.080 |  |
| Mid-Range Latitude (°) | 0.5920 | -0.3093 | 1.5246 | 0.204 |  |

**Table S97.** Predictors of colourful head pelage within Order Cetartiodactyla.

| **Fixed Effects** | Z-score | Lower 95% CI | Upper 95% CI | pMCMC |
| --- | --- | --- | --- | --- |
| Temperature (°) | 0.4107 | -0.3406 | 1.1475 | 0.228 |
| PC1 | 0.2656 | -0.3714 | 0.8587 | 0.338 |
| Mid-Range Latitude (°) | -0.3235 | -1.1799 | 0.4861 | 0.390 |

**Table S98.** Predictors of colourful head pelage within Order Dasyuromorphia.

| **Fixed Effects** | Z-score | Lower 95% CI | Upper 95% CI | pMCMC |
| --- | --- | --- | --- | --- |
| Temperature (°) | 1.4188 | -1.6849 | 4.3912 | 0.336 |
| PC1 | 0.3059 | -2.9818 | 3.4782 | 0.794 |
| Mid-Range Latitude (°) | 0.6978 | -2.5501 | 3.8621 | 0.620 |

**Table S99.** Predictors of colourful head pelage within Order Diprotodontia.

| **Fixed Effects** | Z-score | Lower 95% CI | Upper 95% CI | pMCMC |
| --- | --- | --- | --- | --- |
| Temperature (°) | -1.8774 | -4.2956 | 0.5296 | 0.080 |
| PC1 | -0.5286 | -2.6867 | 1.3857 | 0.524 |
| Mid-Range Latitude (°) | -1.4640 | -4.0822 | 0.9908 | 0.240 |

**Table S100.** Predictors of colourful head pelage within Order Eulipotyphla.

| **Fixed Effects** | Z-score | Lower 95% CI | Upper 95% CI | pMCMC |
| --- | --- | --- | --- | --- |
| Temperature (°) | 0.0392 | -0.5403 | 0.7084 | 0.900 |
| Mean Precipitation (mm/m) | -0.3087 | -1.5250 | 1.0268 | 0.624 |
| Mean Evapotranspiration (mm/m) | -0.8342 | -2.4616 | 0.7731 | 0.262 |
| Mid-Range Latitude (°) | -0.224 | -0.8470 | 0.9820 | 0.930 |

**Table S101.** Predictors of colourful head pelage within Order Primates.

| **Fixed Effects** | Z-score | Lower 95% CI | Upper 95% CI | pMCMC |
| --- | --- | --- | --- | --- |
| Temperature (°) | -0.3553 | -2.1316 | 1.0973 | 0.706 |
| Mean Precipitation (mm/m) | 0.3199 | -1.2312 | 2.0007 | 0.684 |
| Mean Evapotranspiration (mm/m) | -0.0582 | -2.0328 | 1.8001 | 0.958 |
| Mid-Range Latitude (°) | 0.4440 | -0.7105 | 1.6548 | 0.430 |

**Table S102.** Predictors of colourful tail pelage across the mammal Class.

| **Fixed Effects** | Z-score | Lower 95% CI | Upper 95% CI | pMCMC |
| --- | --- | --- | --- | --- |
| Temperature (°) | -0.0796 | -0.2640 | 0.1208 | 0.442 |
| PC1 | -0.0162 | -0.1618 | 0.1358 | 0.836 |
| Mid-Range Latitude (°) | 0.0079 | -0.1692 | 0.1990 | 0.964 |

**Table S103.** Predictors of colourful tail pelage within Order Carnivora.

| **Fixed Effects** | Z-score | Lower 95% CI | Upper 95% CI | pMCMC |
| --- | --- | --- | --- | --- |
| Temperature (°) | 0.2674 | -0.4520 | 1.0203 | 0.414 |
| PC1 | -0.0540 | -0.6546 | 0.5744 | 0.864 |
| Mid-Range Latitude (°) | 0.3540 | -0.2407 | 1.0345 | 0.282 |

**Table S104.** Predictors of colourful tail pelage within Order Cetartiodactyla.

| **Fixed Effects** | Z-score | Lower 95% CI | Upper 95% CI | pMCMC |
| --- | --- | --- | --- | --- |
| Temperature (°) | -0.7009 | -2.9839 | 0.8414 | 0.388 |
| PC1 | 0.1582 | -1.1999 | 1.5582 | 0.854 |
| Mid-Range Latitude (°) | -0.0679 | -1.7174 | 1.5110 | 0.912 |

**Table S105.** Predictors of colourful tail pelage within Order Dasyuromorphia.

| **Fixed Effects** | Z-score | Lower 95% CI | Upper 95% CI | pMCMC |
| --- | --- | --- | --- | --- |
| Temperature (°) | -0.6052 | -3.4403 | 2.7017 | 0.724 |
| PC1 | 1.0672 | -2.6765 | 4.0616 | 0.476 |
| Mid-Range Latitude (°) | 0.9208 | -2.3044 | 3.7648 | 0.528 |

**Table S106.** Predictors of colourful tail pelage within Order Diprotodontia.

| **Fixed Effects** | Z-score | Lower 95% CI | Upper 95% CI | pMCMC |  |
| --- | --- | --- | --- | --- | --- |
| Temperature (°) | | 0.8568 | -1.1239 | 3.0704 | 0.372 |
| PC1 | -1.7646 | -4.0803 | 0.1420 | 0.074 |  |
| Mid-Range Latitude (°) | -0.8267 | -3.2657 | 1.2403 | 0.408 |  |

**Table S107.** Predictors of colourful tail pelage within Order Eulipotyphla.

| **Fixed Effects** | Z-score | Lower 95% CI | Upper 95% CI | pMCMC |  |
| --- | --- | --- | --- | --- | --- |
| Temperature (°) | | -0.8691 | -2.0665 | 0.0781 | 0.058 |
| Mean Precipitation (mm/m) | 0.4315 | -1.1244 | 2.5701 | 0.634 |  |
| Mean Evapotranspiration (mm/m) | -0.2010 | -2.5298 | 1.8641 | 0.862 |  |
| Mid-Range Latitude (°) | 1.6710 | -0.1855 | 3.9499 | 0.068 |  |

**Table S108.** Predictors of colourful tail pelage within Order Rodentia.

| **Fixed Effects** | Z-score | Lower 95% CI | Upper 95% CI | pMCMC |
| --- | --- | --- | --- | --- |
| Temperature (°) | -0.1172 | -0.4159 | 0.1765 | 0.440 |
| PC1 | 0.0210 | -0.2055 | 0.2502 | 0.868 |
| Mid-Range Latitude (°) | 0.0652 | -0.2272 | 0.3671 | 0.636 |

Non-significant contrasting pelage results

**Table S109.** Predictors of contrasting torso pelage within Order Carnivora.

| **Fixed Effects** | Z-score | Lower 95% CI | Upper 95% CI | pMCMC |
| --- | --- | --- | --- | --- |
| Temperature (°) | -0.2629 | -1.2850 | 0.5000 | 0.546 |
| PC1 | 0.0585 | -0.6060 | 0.6477 | 0.850 |
| Mid-Range Latitude (°) | 0.1896 | -0.4505 | 0.8843 | 0.520 |

**Table S110.** Predictors of contrasting torso pelage within Order Cetartiodactyla.

| **Fixed Effects** | Z-score | Lower 95% CI | Upper 95% CI | pMCMC |
| --- | --- | --- | --- | --- |
| Temperature (°) | -2.6714 | -5.6188 | -0.1759 | 0.050 |
| PC1 | 1.2008 | -0.1388 | 3.0389 | 0.078 |
| Mid-Range Latitude (°) | 0.7103 | -0.7832 | 2.5389 | 0.306 |

**Table S111.** Predictors of contrasting torso pelage within Order Diprotodontia.

| **Fixed Effects** | Z-score | Lower 95% CI | Upper 95% CI | pMCMC |
| --- | --- | --- | --- | --- |
| Temperature (°) | -1.0521 | -3.0773 | 0.6170 | 0.202 |
| PC1 | -0.1085 | -1.7539 | 1.4076 | 0.850 |
| Mid-Range Latitude (°) | -1.4987 | -3.9416 | 0.6714 | 0.158 |

**Table S112.** Predictors of contrasting torso pelage within Order Eulipotyphla.

| **Fixed Effects** | Z-score | Lower 95% CI | Upper 95% CI | pMCMC |
| --- | --- | --- | --- | --- |
| Temperature (°) | 0.3410 | -1.4868 | 1.8622 | 0.614 |
| Mean Precipitation (mm/m) | -1.6412 | -4.0818 | 0.5207 | 0.142 |
| Mean Evapotranspiration (mm/m) | 0.2950 | -2.4591 | 2.8462 | 0.784 |
| Mid-Range Latitude (°) | 0.8913 | -0.7146 | 3.3176 | 0.282 |

**Table S113.** Predictors of contrasting torso pelage within Order Primates.

| **Fixed Effects** | Z-score | Lower 95% CI | Upper 95% CI | pMCMC |
| --- | --- | --- | --- | --- |
| Temperature (°) | -0.1261 | -0.9418 | 0.6372 | 0.854 |
| Mean Precipitation (mm/m) | 0.5615 | -0.4688 | 1.4676 | 0.236 |
| Mean Evapotranspiration (mm/m) | -0.8998 | -2.0831 | 0.2097 | 0.096 |
| Mid-Range Latitude (°) | -0.1137 | -0.7512 | 0.6559 | 0.754 |

**Table S114.** Predictors of contrasting torso pelage within Order Rodentia.

| **Fixed Effects** | Z-score | Lower 95% CI | Upper 95% CI | pMCMC |  |
| --- | --- | --- | --- | --- | --- |
| Temperature (°) | | -0.1896 | -0.5444 | 0.1303 | 0.276 |
| PC1 | 0.0898 | -0.1258 | 0.3093 | 0.416 |  |
| Mid-Range Latitude (°) | 0.2244 | -0.0417 | 0.4912 | 0.092 |  |

**Table S115.** Predictors of contrasting leg pelage within Order Carnivora.

| **Fixed Effects** | Z-score | Lower 95% CI | Upper 95% CI | pMCMC |
| --- | --- | --- | --- | --- |
| Temperature (°) | 0.2290 | -0.3863 | 0.8335 | 0.436 |
| PC1 | -0.4015 | -1.0761 | 0.1974 | 0.224 |
| Mid-Range Latitude (°) | 0.5299 | -0.1083 | 1.1474 | 0.094 |

**Table S116.** Predictors of contrasting leg pelage within Order Cetartiodactyla.

| **Fixed Effects** | Z-score | Lower 95% CI | Upper 95% CI | pMCMC |
| --- | --- | --- | --- | --- |
| Temperature (°) | -0.5353 | -2.5767 | 1.0334 | 0.502 |
| PC1 | 0.9596 | -0.2178 | 2.4233 | 0.112 |
| Mid-Range Latitude (°) | -1.4110 | -3.4334 | 0.5183 | 0.078 |

**Table S117.** Predictors of contrasting leg pelage within Order Diprotodontia.

| **Fixed Effects** | Z-score | Lower 95% CI | Upper 95% CI | pMCMC |
| --- | --- | --- | --- | --- |
| Temperature (°) | 0.5478 | -2.0031 | 3.6570 | 0.720 |
| PC1 | -1.0777 | -4.1707 | 1.6688 | 0.440 |
| Mid-Range Latitude (°) | -1.4416 | -4.6691 | 1.9244 | 0.378 |

**Table S118.** Predictors of contrasting leg pelage within Order Primates.

| **Fixed Effects** | Z-score | Lower 95% CI | Upper 95% CI | pMCMC |
| --- | --- | --- | --- | --- |
| Temperature (°) | -0.5369 | -1.5933 | 0.3484 | 0.272 |
| Mean Precipitation (mm/m) | -0.9187 | -1.8521 | 0.0919 | 0.068 |
| Mean Evapotranspiration (mm/m) | 0.2343 | -0.7674 | 1.4070 | 0.658 |
| Mid-Range Latitude (°) | 0.3298 | -0.3850 | 1.2170 | 0.376 |

**Table S119.** Predictors of contrasting head pelage across the mammal Class.

| **Fixed Effects** | Z-score | Lower 95% CI | Upper 95% CI | pMCMC |
| --- | --- | --- | --- | --- |
| Temperature (°) | -0.0664 | -0.2615 | 0.1342 | 0.520 |
| PC1 | 0.1256 | -0.0048 | 0.2868 | 0.094 |
| Mid-Range Latitude (°) | 0.0064 | -0.1715 | 0.2167 | 0.942 |

**Table S120.** Predictors of contrasting head pelage within Order Carnivora.

| **Fixed Effects** | Z-score | Lower 95% CI | Upper 95% CI | pMCMC |
| --- | --- | --- | --- | --- |
| Temperature (°) | -0.6971 | -1.7256 | 0.2263 | 0.158 |
| PC1 | -0.1119 | -0.6716 | 0.4676 | 0.748 |
| Mid-Range Latitude (°) | -0.3546 | -1.0337 | 0.2554 | 0.264 |

**Table S121.** Predictors of contrasting head pelage within Order Cetartiodactyla.

| **Fixed Effects** | Z-score | Lower 95% CI | Upper 95% CI | pMCMC |
| --- | --- | --- | --- | --- |
| Temperature (°) | 0.0093 | -0.6767 | 0.6687 | 0.976 |
| PC1 | 0.0986 | -0.4403 | 0.7098 | 0.726 |
| Mid-Range Latitude (°) | 0.0339 | -0.5708 | 0.6855 | 0.872 |

**Table S122.** Predictors of contrasting head pelage within Order Diprotodontia.

| **Fixed Effects** | Z-score | Lower 95% CI | Upper 95% CI | pMCMC |  |
| --- | --- | --- | --- | --- | --- |
| Temperature (°) | | -0.0218 | -1.0240 | 0.9319 | 0.980 |
| PC1 | -0.2859 | -1.2421 | 0.7390 | 0.544 |  |
| Mid-Range Latitude (°) | -1.2141 | -3.0421 | 0.0443 | 0.060 |  |

**Table S123.** Predictors of contrasting head pelage within Order Eulipotyphla.

| **Fixed Effects** | Z-score | Lower 95% CI | Upper 95% CI | pMCMC |
| --- | --- | --- | --- | --- |
| Temperature (°) | 0.6837 | -1.3852 | 2.8087 | 0.470 |
| Mean Precipitation (mm/m) | -1.8654 | -4.4705 | 1.2634 | 0.208 |
| Mean Evapotranspiration (mm/m) | -0.3184 | -3.0748 | 2.7709 | 0.820 |
| Mid-Range Latitude (°) | -1.9630 | -4.6204 | 0.8937 | 0.142 |

**Table S124.** Predictors of contrasting head pelage within Order Primates.

| **Fixed Effects** | Z-score | Lower 95% CI | Upper 95% CI | pMCMC |
| --- | --- | --- | --- | --- |
| Temperature (°) | -0.0094 | -0.6254 | 0.6114 | 0.936 |
| Mean Precipitation (mm/m) | -0.7396 | -1.6777 | 0.2296 | 0.130 |
| Mean Evapotranspiration (mm/m) | 0.5033 | -0.6171 | 1.6036 | 0.368 |
| Mid-Range Latitude (°) | 0.0647 | -0.7379 | 0.9517 | 0.850 |

**Table S125.** Predictors of contrasting tail pelage across the mammal Class.

| **Fixed Effects** | Z-score | Lower 95% CI | Upper 95% CI | pMCMC |
| --- | --- | --- | --- | --- |
| Temperature (°) | -0.1087 | -0.3721 | 0.1715 | 0.442 |
| PC1 | 0.1208 | -0.1053 | 0.3327 | 0.270 |
| Mid-Range Latitude (°) | 0.1727 | -0.0666 | 0.4533 | 0.188 |

**Table S126.** Predictors of contrasting tail pelage within Order Carnivora.

| **Fixed Effects** | Z-score | Lower 95% CI | Upper 95% CI | pMCMC |
| --- | --- | --- | --- | --- |
| Temperature (°) | -1.0993 | -2.9896 | 0.4206 | 0.162 |
| PC1 | -0.4604 | -1.8186 | 0.9994 | 0.418 |
| Mid-Range Latitude (°) | -0.1200 | -1.2184 | 1.1105 | 0.818 |

**Table S127.** Predictors of contrasting tail pelage within Order Cetartiodactyla.

| **Fixed Effects** | Z-score | Lower 95% CI | Upper 95% CI | pMCMC |
| --- | --- | --- | --- | --- |
| Temperature (°) | -0.6590 | -3.7493 | 2.5735 | 0.674 |
| PC1 | 1.4690 | -0.3139 | 3.5153 | 0.074 |
| Mid-Range Latitude (°) | -1.4086 | -4.8153 | 1.5681 | 0.356 |

**Table S128.** Predictors of contrasting tail pelage within Order Diprotodontia.

| **Fixed Effects** | Z-score | Lower 95% CI | Upper 95% CI | pMCMC |
| --- | --- | --- | --- | --- |
| Temperature (°) | -0.7303 | -3.1965 | 1.1131 | 0.374 |
| PC1 | 0.7082 | -1.3982 | 3.1326 | 0.418 |
| Mid-Range Latitude (°) | 0.0534 | -2.3393 | 2.7687 | 0.986 |

**Table S129.** Predictors of contrasting tail pelage within Order Primates.

| **Fixed Effects** | Z-score | Lower 95% CI | Upper 95% CI | pMCMC |
| --- | --- | --- | --- | --- |
| Temperature (°) | -0.2141 | -2.2374 | 1.5180 | 0.790 |
| Mean Precipitation (mm/m) | 0.5985 | -1.2831 | 2.9303 | 0.558 |
| Mean Evapotranspiration (mm/m) | -0.6647 | -2.9225 | 1.3304 | 0.502 |
| Mid-Range Latitude (°) | 0.4683 | -1.1585 | 2.7841 | 0.548 |

**Table S130.** Predictors of contrasting tail pelage within Order Rodentia.

| **Fixed Effects** | Z-score | Lower 95% CI | Upper 95% CI | pMCMC |
| --- | --- | --- | --- | --- |
| Temperature (°) | -0.2014 | -0.5569 | 0.1791 | 0.252 |
| PC1 | 0.0992 | -0.1831 | 0.3882 | 0.512 |
| Mid-Range Latitude (°) | 0.1642 | -0.1620 | 0.5385 | 0.322 |

Non-significant patterned pelage results

**Table S131.** Predictors of patterned torso coloration within Order Carnivora.

| **Fixed Effects** | Z-score | Lower 95% CI | Upper 95% CI | pMCMC |
| --- | --- | --- | --- | --- |
| Temperature (°) | 0.0452 | -0.6521 | 0.7183 | 0.904 |
| PC1 | -0.2133 | -0.8289 | 0.3818 | 0.510 |
| Mid-Range Latitude (°) | 0.0965 | -0.6580 | 0.7647 | 0.734 |

**Table S132.** Predictors of patterned torso coloration within Order Cetartiodactyla.

| **Fixed Effects** | Z-score | Lower 95% CI | Upper 95% CI | pMCMC |
| --- | --- | --- | --- | --- |
| Temperature (°) | 0.1791 | -0.7750 | 1.3235 | 0.734 |
| PC1 | -0.0816 | -1.0480 | 0.8103 | 0.940 |
| Mid-Range Latitude (°) | 0.0238 | -1.0967 | 1.0180 | 0.962 |

**Table S133.** Predictors of patterned torso coloration within Order Dasyuromorphia.

| **Fixed Effects** | Z-score | Lower 95% CI | Upper 95% CI | pMCMC |
| --- | --- | --- | --- | --- |
| Temperature (°) | 1.0840 | -1.1830 | 3.8780 | 0.352 |
| PC1 | 1.2840 | -1.2780 | 3.8880 | 0.270 |
| Mid-Range Latitude (°) | 1.2270 | -1.6460 | 3.6400 | 0.298 |

**Table S134.** Predictors of patterned torso coloration within Order Diprotodontia.

| **Fixed Effects** | Z-score | Lower 95% CI | Upper 95% CI | pMCMC |
| --- | --- | --- | --- | --- |
| Temperature (°) | -1.7282 | -4.2824 | 0.3737 | 0.116 |
| PC1 | -0.7925 | -2.9060 | 1.4329 | 0.440 |
| Mid-Range Latitude (°) | -0.4220 | -2.9038 | 2.1188 | 0.696 |

**Table S135.** Predictors of patterned torso coloration within Order Eulipotyphla.

| **Fixed Effects** | Z-score | Lower 95% CI | Upper 95% CI | pMCMC |
| --- | --- | --- | --- | --- |
| Temperature (°) | -0.3998 | -1.1151 | 0.2604 | 0.232 |
| Mean Precipitation (mm/m) | -0.7406 | -1.7647 | 0.4349 | 0.184 |
| Mean Evapotranspiration (mm/m) | -0.2283 | -1.3642 | 1.0434 | 0.720 |
| Mid-Range Latitude (°) | -0.1691 | -0.7041 | 0.4241 | 0.538 |

**Table S136.** Predictors of patterned leg coloration across the mammal Class.

| **Fixed Effects** | Z-score | Lower 95% CI | Upper 95% CI | pMCMC |
| --- | --- | --- | --- | --- |
| Temperature (°) | 0.1560 | -0.0556 | 0.3639 | 0.146 |
| PC1 | -0.0163 | -0.1670 | 0.1416 | 0.846 |
| Mid-Range Latitude (°) | -0.0397 | -0.2374 | 0.1535 | 0.664 |

**Table S137.** Predictors of patterned leg coloration within Order Carnivora.

| **Fixed Effects** | Z-score | Lower 95% CI | Upper 95% CI | pMCMC |
| --- | --- | --- | --- | --- |
| Temperature (°) | 0.0554 | -1.3466 | 1.8580 | 0.934 |
| PC1 | -0.8040 | -2.2004 | 0.3077 | 0.180 |
| Mid-Range Latitude (°) | 0.6590 | -0.6632 | 2.0876 | 0.276 |

**Table S138.** Predictors of patterned leg coloration within Order Cetartiodactyla.

| **Fixed Effects** | Z-score | Lower 95% CI | Upper 95% CI | pMCMC |
| --- | --- | --- | --- | --- |
| Temperature (°) | 0.3683 | -0.2840 | 1.1818 | 0.294 |
| PC1 | 0.1863 | -0.3574 | 0.6484 | 0.454 |
| Mid-Range Latitude (°) | -0.2524 | -0.9592 | 0.5306 | 0.500 |

**Table S139.** Predictors of patterned leg coloration within Order Dasyuromorphia.

| **Fixed Effects** | Z-score | Lower 95% CI | Upper 95% CI | pMCMC |
| --- | --- | --- | --- | --- |
| Temperature (°) | -0.1991 | -3.1274 | 2.8298 | 0.932 |
| PC1 | 1.4727 | -1.4540 | 4.7314 | 0.310 |
| Mid-Range Latitude (°) | -0.3846 | -3.4907 | 2.5128 | 0.816 |

**Table S140.** Predictors of patterned leg coloration within Order Diprotodontia.

| **Fixed Effects** | Z-score | Lower 95% CI | Upper 95% CI | pMCMC |
| --- | --- | --- | --- | --- |
| Temperature (°) | 0.8824 | -0.8565 | 2.9819 | 0.336 |
| PC1 | -1.4998 | -3.8622 | 0.4632 | 0.102 |
| Mid-Range Latitude (°) | 1.2730 | -0.8178 | 3.7142 | 0.216 |

**Table S141.** Predictors of patterned leg coloration within Order Eulipotyphla.

| **Fixed Effects** | Z-score | Lower 95% CI | Upper 95% CI | pMCMC |
| --- | --- | --- | --- | --- |
| Temperature (°) | 0.4547 | -1.1866 | 2.6209 | 0.720 |
| Mean Precipitation (mm/m) | 0.7008 | -1.5843 | 3.1218 | 0.554 |
| Mean Evapotranspiration (mm/m) | -0.5084 | -3.0504 | 2.3907 | 0.732 |
| Mid-Range Latitude (°) | -0.0778 | -1.7429 | 1.3099 | 0.908 |

**Table S142.** Predictors of patterned leg coloration within Order Primates.

| **Fixed Effects** | Z-score | Lower 95% CI | Upper 95% CI | pMCMC |
| --- | --- | --- | --- | --- |
| Temperature (°) | -0.2219 | -0.6090 | 0.2034 | 0.280 |
| Mean Precipitation (mm/m) | -0.0144 | -0.5438 | 0.5485 | 0.990 |
| Mean Evapotranspiration (mm/m) | 0.2973 | -0.3694 | 0.8980 | 0.378 |
| Mid-Range Latitude (°) | -0.0249 | -0.4199 | 0.3915 | 0.928 |

**Table S143.** Predictors of patterned leg coloration within Order Rodentia.

| **Fixed Effects** | Z-score | Lower 95% CI | Upper 95% CI | pMCMC |
| --- | --- | --- | --- | --- |
| Temperature (°) | 0.2989 | 0.0033 | 0.6607 | 0.070 |
| PC1 | -0.0903 | -0.3319 | 0.1573 | 0.488 |
| Mid-Range Latitude (°) | -0.0379 | -0.3456 | 0.3008 | 0.846 |

**Table S144.** Predictors of patterned head coloration within Order Carnivora.

| **Fixed Effects** | Z-score | Lower 95% CI | Upper 95% CI | pMCMC |  |
| --- | --- | --- | --- | --- | --- |
| Temperature (°) | | -1.6315 | -3.9962 | 0.4015 | 0.124 |
| PC1 | -1.1302 | -2.4704 | 0.1459 | 0.066 |  |
| Mid-Range Latitude (°) | 0.5644 | -0.2793 | 1.4877 | 0.192 |  |

**Table S145.** Predictors of patterned head coloration within Order Cetartiodactyla.

| **Fixed Effects** | Z-score | Lower 95% CI | Upper 95% CI | pMCMC |
| --- | --- | --- | --- | --- |
| Temperature (°) | 0.3431 | -0.6838 | 1.1865 | 0.374 |
| PC1 | 0.2428 | -0.5979 | 0.9968 | 0.450 |
| Mid-Range Latitude (°) | -0.4516 | -1.6968 | 0.5690 | 0.370 |

**Table S146.** Predictors of patterned head coloration within Order Dasyuromorphia.

| **Fixed Effects** | Z-score | Lower 95% CI | Upper 95% CI | pMCMC |
| --- | --- | --- | --- | --- |
| Temperature (°) | 1.4331 | -1.5435 | 4.5132 | 0.358 |
| PC1 | 0.3696 | -2.9466 | 3.4385 | 0.782 |
| Mid-Range Latitude (°) | 0.6117 | -2.5932 | 3.9205 | 0.674 |

**Table S147.** Predictors of patterned head coloration within Order Diprotodontia.

| **Fixed Effects** | Z-score | Lower 95% CI | Upper 95% CI | pMCMC |
| --- | --- | --- | --- | --- |
| Temperature (°) | -1.8009 | -4.2777 | 0.1859 | 0.078 |
| PC1 | -0.4848 | -2.3561 | 1.3099 | 0.546 |
| Mid-Range Latitude (°) | -1.4600 | -4.1077 | 0.9963 | 0.242 |

**Table S148.** Predictors of patterned head coloration within Order Eulipotyphla.

| **Fixed Effects** | Z-score | Lower 95% CI | Upper 95% CI | pMCMC |
| --- | --- | --- | --- | --- |
| Temperature (°) | 0.0532 | -0.5749 | 0.7287 | 0.820 |
| Mean Precipitation (mm/m) | -0.2990 | -1.6140 | 0.9979 | 0.652 |
| Mean Evapotranspiration (mm/m) | -0.8809 | -2.5436 | 0.5805 | 0.204 |
| Mid-Range Latitude (°) | -0.0583 | -0.9992 | 0.7841 | 0.822 |

**Table S149.** Predictors of patterned head coloration within Order Primates.

| **Fixed Effects** | Z-score | Lower 95% CI | Upper 95% CI | pMCMC |
| --- | --- | --- | --- | --- |
| Temperature (°) | -0.4399 | -2.1113 | 1.2122 | 0.586 |
| Mean Precipitation (mm/m) | 0.2682 | -1.3786 | 1.8557 | 0.754 |
| Mean Evapotranspiration (mm/m) | -0.0372 | -1.9385 | 1.9922 | 0.972 |
| Mid-Range Latitude (°) | 0.4317 | -0.8232 | 1.6986 | 0.480 |

**Table S150.** Predictors of patterned tail coloration across the mammal Class.

| **Fixed Effects** | Z-score | Lower 95% CI | Upper 95% CI | pMCMC |
| --- | --- | --- | --- | --- |
| Temperature (°) | -0.0767 | -0.2791 | 0.1298 | 0.460 |
| PC1 | -0.0203 | -0.1789 | 0.1373 | 0.798 |
| Mid-Range Latitude (°) | 0.0104 | -0.2222 | 0.1867 | 0.896 |

**Table S151.** Predictors of patterned tail coloration within Order Carnivora.

| **Fixed Effects** | Z-score | Lower 95% CI | Upper 95% CI | pMCMC |
| --- | --- | --- | --- | --- |
| Temperature (°) | 0.2804 | -0.4080 | 0.9745 | 0.388 |
| PC1 | -0.0363 | -0.6484 | 0.5412 | 0.930 |
| Mid-Range Latitude (°) | 0.3578 | -0.2602 | 0.9595 | 0.252 |

**Table S152.** Predictors of patterned tail coloration within Order Cetartiodactyla.

| **Fixed Effects** | Z-score | Lower 95% CI | Upper 95% CI | pMCMC |
| --- | --- | --- | --- | --- |
| Temperature (°) | -0.7182 | -3.2083 | 0.7800 | 0.430 |
| PC1 | 0.1383 | -1.1161 | 1.3969 | 0.848 |
| Mid-Range Latitude (°) | -0.0560 | -1.9609 | 1.2628 | 0.944 |

**Table S153.** Predictors of patterned tail coloration within Order Dasyuromorphia.

| **Fixed Effects** | Z-score | Lower 95% CI | Upper 95% CI | pMCMC |
| --- | --- | --- | --- | --- |
| Temperature (°) | -1.1141 | -4.1298 | 1.6029 | 0.432 |
| PC1 | 0.6352 | -2.7559 | 3.8553 | 0.654 |
| Mid-Range Latitude (°) | 1.1644 | -1.7750 | 4.1705 | 0.432 |

**Table S154.** Predictors of patterned tail coloration within Order Diprotodontia.

| **Fixed Effects** | Z-score | Lower 95% CI | Upper 95% CI | pMCMC |  |
| --- | --- | --- | --- | --- | --- |
| Temperature (°) | | 0.8549 | -0.9970 | 3.0348 | 0.368 |
| PC1 | -1.8144 | -4.0521 | 0.4814 | 0.080 |  |
| Mid-Range Latitude (°) | -0.8345 | -2.9282 | 1.6276 | 0.378 |  |

**Table S155.** Predictors of patterned tail coloration within Order Eulipotyphla.

| **Fixed Effects** | Z-score | Lower 95% CI | Upper 95% CI | pMCMC |  |
| --- | --- | --- | --- | --- | --- |
| Temperature (°) | | -0.8424 | -1.9907 | 0.0662 | 0.066 |
| Mean Precipitation (mm/m) | 0.4432 | -1.1373 | 2.3546 | 0.638 |  |
| Mean Evapotranspiration (mm/m) | -0.2584 | -2.3698 | 1.6375 | 0.836 |  |
| Mid-Range Latitude (°) | 1.5580 | -0.5525 | 3.6186 | 0.110 |  |

**Table S156.** Predictors of patterned tail coloration within Order Rodentia.

| **Fixed Effects** | Z-score | Lower 95% CI | Upper 95% CI | pMCMC |
| --- | --- | --- | --- | --- |
| Temperature (°) | -0.1271 | -0.4285 | 0.1689 | 0.394 |
| PC1 | 0.0152 | -0.2243 | 0.2265 | 0.912 |
| Mid-Range Latitude (°) | 0.0623 | -0.2023 | 0.3658 | 0.690 |
